# Reengineering Redox Sensitive GFP to Measure Mycothiol Redox Potential of *Mycobacterium tuberculosis* during Infection

**DOI:** 10.1371/journal.ppat.1003902

**Published:** 2014-01-30

**Authors:** Ashima Bhaskar, Manbeena Chawla, Mansi Mehta, Pankti Parikh, Pallavi Chandra, Devayani Bhave, Dhiraj Kumar, Kate S. Carroll, Amit Singh

**Affiliations:** 1 International Centre for Genetic Engineering and Biotechnology, New Delhi, India; 2 Department of Chemistry, The Scripps Research Institute, Jupiter, Florida, United States of America; University of Massachusetts, United States of America

## Abstract

*Mycobacterium tuberculosis (Mtb)* survives under oxidatively hostile environments encountered inside host phagocytes. To protect itself from oxidative stress, *Mtb* produces millimolar concentrations of mycothiol (MSH), which functions as a major cytoplasmic redox buffer. Here, we introduce a novel system for real-time imaging of mycothiol redox potential (*E_MSH_*) within *Mtb* cells during infection. We demonstrate that coupling of *Mtb* MSH-dependent oxidoreductase (mycoredoxin-1; Mrx1) to redox-sensitive GFP (roGFP2; Mrx1-roGFP2) allowed measurement of dynamic changes in intramycobacterial *E_MSH_* with unprecedented sensitivity and specificity. Using Mrx1-roGFP2, we report the first quantitative measurements of *E_MSH_* in diverse mycobacterial species, genetic mutants, and drug-resistant patient isolates. These cellular studies reveal, for the first time, that the environment inside macrophages and sub-vacuolar compartments induces heterogeneity in *E_MSH_* of the *Mtb* population. Further application of this new biosensor demonstrates that treatment of *Mtb* infected macrophage with anti-tuberculosis (TB) drugs induces oxidative shift in *E_MSH_*, suggesting that the intramacrophage milieu and antibiotics cooperatively disrupt the MSH homeostasis to exert efficient *Mtb* killing. Lastly, we analyze the membrane integrity of *Mtb* cells with varied *E_MSH_* during infection and show that subpopulation with higher *E_MSH_* are susceptible to clinically relevant antibiotics, whereas lower *E_MSH_* promotes antibiotic tolerance. Together, these data suggest the importance of MSH redox signaling in modulating mycobacterial survival following treatment with anti-TB drugs. We anticipate that Mrx1-roGFP2 will be a major contributor to our understanding of redox biology of *Mtb* and will lead to novel strategies to target redox metabolism for controlling *Mtb* persistence.

## Author Summary

Approximately 30% of the global population is infected with *Mycobacterium tuberculosis* (*Mtb*). Persistence of *Mtb* in host phagocytes depends on its ability to resist oxidant-mediated antibacterial responses. Mycothiol (MSH) is the main antioxidant that provides an abundant source of reducing equivalent, which protects *Mtb* from oxidative stress encountered during infection. The majority of research into redox signaling in *Mtb* has relied on chemical analysis of MSH in whole cell extract, which creates oxidation artifacts and prohibits dynamic imaging of MSH redox state during infection. We have successfully developed a novel and noninvasive tool based on genetically encoded redox sensitive fluorescent probes to perform real-time measurement of mycothiol redox potential (*E_MSH_*) in *Mtb* during infection. For the first time we reveal the *E_MSH_* of virulent and avirulent mycobacterial strains, including drug-resistant clinical isolates. We used this technology and came to the surprising conclusion that within a single infected macrophage there is heterogeneity in the redox signature of individual *Mtb* bacilli. Importantly, we show that anti-TB drugs accelerate oxidative stress in *Mtb* within infected macrophages and redox heterogeneity can contribute to emergence of drug tolerant population. These findings have implications for mycobacterial persistence following treatment with anti-TB drugs.

## Introduction

It is estimated that nearly 2 billion people currently suffer from latent *Mycobacterium tuberculosis* (*Mtb*) infection and ∼1.4 million people succumb to tuberculosis (TB) annually [1] and [www.who.int/tb]. The ability of *Mtb* to adapt and resist killing by the immune system facilitates its survival, replication, and persistence. Following aerosol exposure, *Mtb* is engulfed by macrophages, and exposed to antimicrobial redox stresses including reactive oxygen and nitrogen species (ROS and RNS) [2]. Mycobacterial killing by ROS and RNS is important for host resistance as demonstrated by the increased susceptibility of NADPH oxidase (NOX2) and nitric oxide synthase (iNOS) deficient mice following *Mtb* challenge [3], [4]. Moreover, children with defective NOX2 suffer from chronic granulomatous disease, are susceptible to TB and even develop severe complications after vaccination with BCG [5]. Consistent with these observations, a recent study elegantly demonstrated the requirement of NADPH oxidase in exerting neutrophil-mediated killing of mycobacteria during infection [6].

In response to oxido-reductive stress, *Mtb* upregulates multiple redox sensing pathways, including SigH/RshA, DosR/S/T, MosR, and the WhiB family, to maintain redox homeostasis [7]–[12]. Furthermore, modified cell wall lipids [13], antioxidant enzymes, and cellular redox buffers, such as MSH, thioredoxins (TRXs), and ergothionine (ERG), assist *Mtb* in mitigating redox stress [14]. Collectively, these studies indicate that dynamic reprogramming of intrabacterial redox metabolism in response to host environment is vital for the persistence of *Mtb*. Despite its recognized importance, tools for monitoring changes in redox state of *Mtb* during infection do not exist. Customary approaches involving NAD^+^/NADH and MSH/MSSM measurements require cell disruption, which precludes real-time analyses, and are plagued by oxidation artifacts. Alternatively, redox-sensitive dyes which are commonly used to detect ROS generation in cells, suffer from non-specificity, irreversibility, and these dyes cannot deliver information regarding the redox potential of a specific redox couple [15], [16]. Therefore, development of a specific, sensitive, and non-invasive technology to study defined redox changes in *Mtb* would contribute significantly to delineating novel redox pathways involved in persistence, drug tolerance, as well as pathogenesis of *Mtb*, and may also have utility in high throughput screens to identify small-molecule modulators of intrabacterial redox homeostasis.

Genetically encoded reduction-oxidation sensitive GFP indicators (roGFPs) have been developed to measure the intracellular glutathione (GSH) redox potential (*E_GSH_*) through interaction with glutaredoxins (Grxs) in many organisms [17]. However, the preference of roGFPs for GSH limits their use in non-GSH producers like gram positive bacteria (e.g. *Bacillus* species, *Staphylococcus aureus*, *Deinococcus radiodurans*) and actinomycetes (e.g. Mycobacteria, Corynebacteria, Streptomyces, Nocardia), which respectively contain bacillithiol (BSH) and MSH as their major redox buffers [18], [19].

Because MSH is functionally analogous to GSH and plays a prominent role in maintaining the reduced state of the mycobacterial cytoplasm [20], [21], we engineered roGFP2 to generate a MSH-specific intracellular probe, Mrx1-roGFP2. Importantly, Mrx1-roGFP2 allows imaging of *E_MSH_* in diverse mycobacterial species and strains, including drug-resistant clinical isolates during infection. Lastly, we examine the potential of antibiotics in inducing intramycobacterial oxidative stress in the physiological context of infection and demonstrate the functional importance of MSH redox signaling in intramacrophage survival and sensitivity to anti-TB drugs. Our study provides an elegant tool to probe redox biology of *Mtb* under diverse environmental conditions including *in vivo* experimental models of TB.

## Results

### Design of the mycothiol-specific fluorescent biosensor

We selected roGFP2 as a fluorescent partner in the biosensor construct because it exhibits the largest dynamic range, it is brighter, pH insensitive, and is resistant to photoswitching [22]. The oxidation of two cysteines on either side of roGFP2 chromophore (S147C and Q204C) [22] generates a disulfide bond and increases the fluorescence intensity at ∼400 nm with concomitant decrease at ∼490 nm, while reduction reverses the spectrum. The 400/490 nm ratio thus reports the redox state of the cell or compartment in which it is expressed [22]. Because the sensor is ratiometric it eliminates errors due to variations in roGFP2 concentrations during different growth phases of an organism.

**Figure 1:**
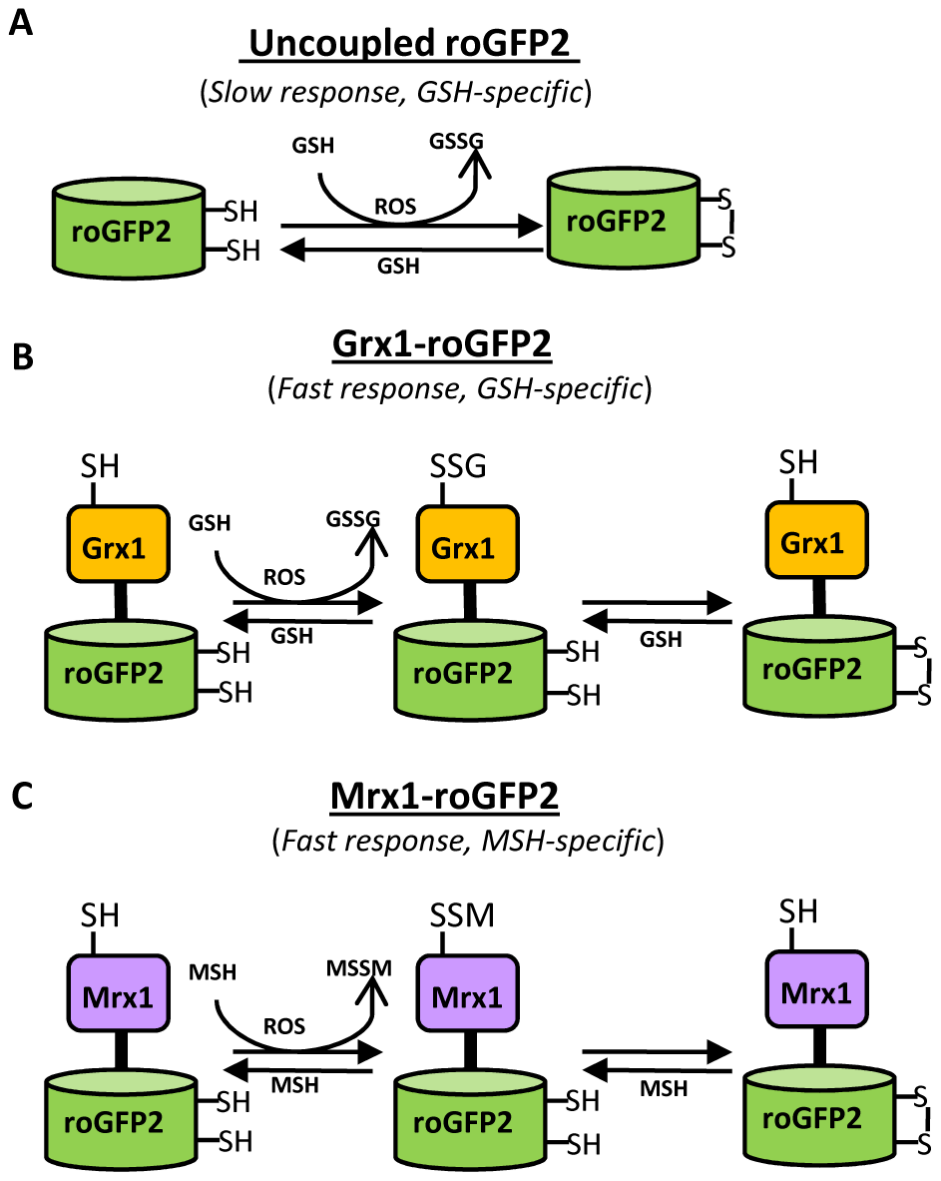
Diagram illustrating the principle of roGFP2 based sensors. (A) roGFP2 contains two Cys residues capable of forming an intramolecular disulfide bond in response to changes in intracellular *E_GSH_*. The redox equilibrium of roGFP2 with the GSH/GSSG couple occurs through endogenous glutaredoxins and proceeds slowly. (B) Fusion of human Grx1 in Grx1-roGFP2 substantially improved specificity and rate of thiol-disulfide exchange between roGFP2 dithiol and 2GSH/GSSG couple. (C) Replacing Grx1 with Mrx1 in Mrx1-roGFP2 ensured continuous equilibration between roGFP2 and 2MSH/MSSM pair.

Conventional roGFP2 predominantly equilibrates with the cytosolic glutathione redox buffer through interaction with endogenous glutaredoxins [23]. However, the response kinetics of roGFP2 was slow and absolute specificity towards GSH/GSSG redox couple cannot be guaranteed [17], [24]. To resolve this, roGFP2 bioprobe was recently fused to human glutaredoxin 1 (Grx1; Grx1-roGFP2), which ensured complete specificity and rapid equilibration with intracellular GSH/GSSG couple [24]. On this basis, we explored the concept of covalently coupling roGFP2 to a mycothiol-specific oxidoreductase such as mycoredoxin (Mrx1) to generate a biosensor (Mrx1-roGFP2) that exclusively responds to perturbations in mycothiol redox potential (*E_MSH_*). The mechanistic basis of coupling Mrx1 to roGFP2 for measuring *E_MSH_* is depicted in Figure 1. Recently, a glutaredoxin (Grx1) homologue (mycoredoxin-1; Mrx1) which exclusively interacts with the mycothiol redox system has been reported in a non-pathogenic saprophytic mycobacteria, *Mycobacterium smegmatis* (*Msm*) [25]. We performed homology based analysis and identified three putative Mrx1 like proteins in *Mtb* H37Rv. Out of the three proteins (Rv3053c, Rv0508, and Rv3198A), Rv3198A demonstrate highest similarity with Mrx1 of *Msm* (72% identity). Based on this, we selected Rv3198A ORF as a putative mycoredoxin-encoding gene and named its product as *Mtb* Mrx1. The *Mtb* Mrx1 contains an active site (CGYC) similar to *Msm* Mrx1, supposedly required for thiol-disulfide exchange activity [25]. We independently replaced the two cysteine (Cys) residues present in the catalytic site of Mrx1 by alanine to generate Mrx1(CGYA) and Mrx1(AGYC). The wt Mrx1 along with its Cys variants were then separately fused to the N-terminus of roGFP2 via a 30-amino acid linker, (Gly-Gly-Ser-Gly-Gly)_6_. The resulting chimeras were affinity purified as His-tagged proteins and analyzed by spectrofluorometry. We observed that Mrx1-roGFP2 exhibits two distinct excitation peaks (390 nm and 490 nm) at a fixed emission wavelength of 510 nm. Therefore, in all subsequent experiments using spectrofluorometer the excitation ratio from these two wavelengths (390 and 490 nm) was measured to determine the extent of biosensor oxidation. Our analysis demonstrated that the intrinsic ratiometric changes exhibited by roGFP2 upon oxidation or reduction were recapitulated in Mrx1-roGFP2 (Figure S1A). In addition, we monitored the response of Mrx1-roGFP2 over a physiologically relevant pH range (pH 5.5–8.5) and found that the fluorescence excitation ratio exhibited by Mrx1-roGFP2 was insensitive to pH variations (Figure S1B). We also verified that the reported midpoint potential (−280 mV) and dynamic range (−320 mV to −240 mV) of roGFP2 were not influenced by the fusion (Figure S1C).

**Figure 2:**
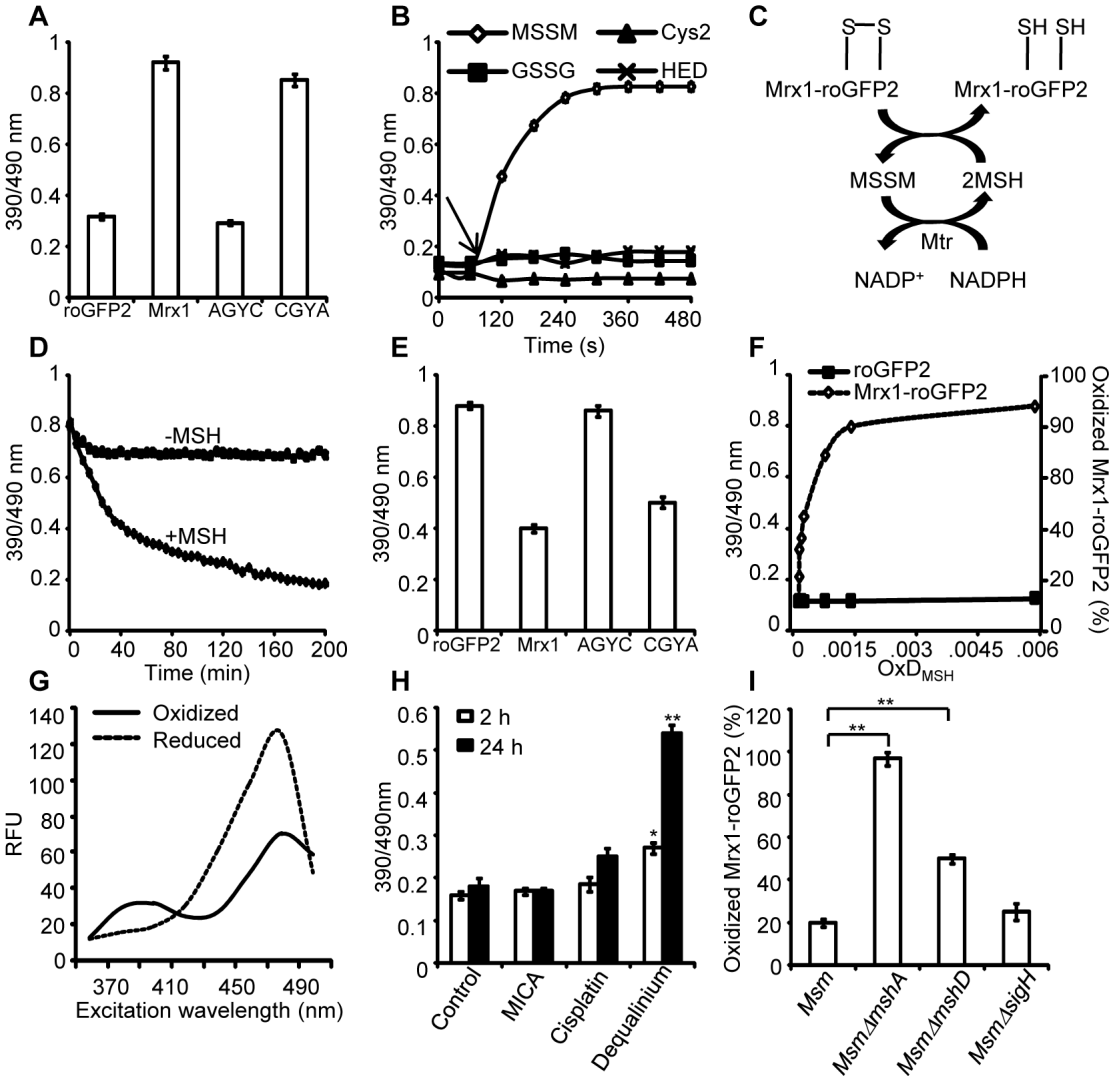
Mrx1 catalyzes specific equilibration between mycothiol redox system and roGFP2 *in vitro* and *in vivo*. (A) Pre-reduced roGFP2 (lane 1), Mrx1-roGFP2 (lane 2), Mrx1(AGYC)-roGFP2 (lane 3), and Mrx1(CGYA)-roGFP2 (lane 4) were exposed to 50 µM of MSSM for 10 min and ratiometric sensor response was measured. (B) Pre-reduced Mrx1-roGFP2 was treated with 1 µM of MSSM, GSSG, cystine (Cys_2_) or 2-hydroxyethyl disulfide (HED) and ratiometric sensor response was measured at various time points. (C) Molecular mechanism showing the reduction of oxidized Mrx1-roGFP2 by MSH/Mtr/NADPH pathway. (D) Oxidized Mrx1-roGFP2 was added as a substrate to the MSH/Mtr/NADPH redox pathway and ratiometric sensor response was measured over time. A control reaction in the absence of MSH was performed in parallel. (E) Reduction of oxidized roGFP2 (lane 1), Mrx1-roGFP2 (lane 2), Mrx1(AGYC)-roGFP2 (lane 3), and Mrx1(CGYA)-roGFP2 (lane 4) by MSH/Mtr/NADPH redox pathway. Maximum ratio change after 150 min of incubation with MSH/Mtr/NADPH reaction mixture is shown. (F) Mrx1-roGFP2 is extremely sensitive towards small changes in OxD_MSH_. Reduced uncoupled roGFP2 and Mrx1-roGFP2 proteins were incubated with mycothiol solutions (1 mM total) containing increasing fractions of MSSM for a maximum of 30 sec and ratiometric sensor response was measured. Note that the response of Mrx1-roGFP2 becomes exceedingly linear in the window between 10% to 90% oxidation, suggesting that the biosensor can effectively measure changes in *E_MSH_* within this range of probe oxidation. (G) Excitation spectra of *Msm* expressing Mrx1-roGFP2 upon treatment with 0.4 mM of diamide (oxidant) or 10 mM of DTT (reductant) for 5 min. (H) *Msm* expressing Mrx1-roGFP2 was either left untreated (control) or exposed to 50 µM dequalinium, cisplatin and 5-methoxyindole-2-carboxylic acid (MICA) and ratiometric sensor response was measured after 2 h and 24 h post-exposure. p-values shown in the panel were calculated by comparing untreated group and dequalinium treated group. (I) Percentage of OxD_Mrx1-roGFP2_ in exponentially grown *Msm*, *MsmΔmshA*, *MsmΔmshD*, and *MsmΔsigH* was calculated (see *Materials and Methods* for mathematical definition). Note that biosensor is completely oxidized (∼95%) in the absence of MSH reducing system in *MsmΔmshA*. p-values shown in the panel were calculated by independently comparing *MsmΔmshA* and *MsmΔmshD* groups with the *Msm* group. Error bars represent standard deviations from the mean. * p<0.01, ** p<0.001. Data shown is the representative of at least three independent experiments.

Since redox stress leads to an increase in oxidized mycothiol (MSSM) concentrations [26], we anticipated that the Mrx1 fusion would enable roGFP2 to selectively monitor this transformation. To test this proposal, we reduced uncoupled roGFP2, Mrx1-roGFP2, Mrx1(CGYA)-roGFP2, and Mrx1(AGYC)-roGFP2 and examined their oxidation by MSSM. Only Mrx1-roGFP2 (Lane 2, Figure 2A) and Mrx1(CGYA)-roGFP2 (Lane 4, Figure 2A) ratios increased upon MSSM addition, whereas uncoupled roGFP2 (Lane 1, Figure 2A) and Mrx1(AGYC)-roGFP2 (Lane 3, Figure 2A) remained non-responsive. This suggests that the Mrx1-roGFP2 reaction mechanism is similar to monothiol mechanism of glutaredoxins, wherein nucleophilic N-terminus cysteine interacts with GSSG to form mixed protein-glutathione disulfide intermediate followed by protein oxidation [24]. Importantly, Mrx1-roGFP2 did not respond to other disulfide-based compounds such as cystine (Cys_2_), GSSG, or 2-hydroxyethyl disulfide (HED), thus confirming the specificity of this biosensor towards MSSM (Figure 2B). Next, we examined the response of Mrx1-roGFP2 to reduced mycothiol (MSH). To continuously maintain a reduced state of MSH in our assays, we used MSH disulfide reductase (Mtr) enzyme. Mtr is known to catalyze the NADPH-dependent reduction of MSSM to MSH in the mycobacterial cells [27]. We first confirmed the activity of purified Mtr by monitoring NADPH oxidation in the presence of MSSM. A time-dependent decrease in 340 nm absorption due to NADPH consumption confirmed cycling of electrons from NADPH to MSSM by Mtr (Figure S1D). Next, oxidized Mrx1-roGFP2 was added as substrate for the MSH/Mtr/NADPH electron transfer assay as depicted in Figure 2C. By monitoring the decrease in the 390/490 excitation ratio, we examined the real-time response of oxidized Mrx1-roGFP2 to MSH generated via NADPH-dependent reduction of MSSM by Mtr. As shown in Figure 2D, a time-dependent decrease in 390/490 ratio confirms reduction of Mrx1-roGFP2 by MSH. The slow response of this biosensor towards MSH is consistent with the low catalytic turnover rate of Mtr [27]. No response was observed if either MSH was omitted from the Mtr/NADPH/Mrx1-roGFP2 mixture (Figure 2D) or catalytically inactive Mrx1(AGYC)-roGFP2 (Lane 3, Figure 2E) or uncoupled roGFP2 (Lane 1, Figure 2E) were used as substrates, whereas a response was readily detected in the case of Mrx1(CGYA)-roGFP2 (Lane 4, Figure 2E). Finally, to measure the sensitivity of the biosensor, we incubated pre-reduced Mrx1-roGFP2 with various ratios of MSH/MSSM at a physiological concentration (1 mM total) under anaerobic conditions and the ratiometric response was monitored. We found that a small increase in MSSM led to a significant increase in biosensor oxidation. For example, an increase in the amount of mycothiol oxidation (OxD_MSH_, see *Materials and Methods* for a mathematical explanation) from 0.00001 to 0.0001 (i. e an ∼100 nM increase in absolute MSSM) led to a larger increase in the biosensor oxidation (i. e from ∼40% to 90%) (Figure 2F). These results confirm that Mrx1-roGFP2 is capable of rapidly sensing nanomolar changes in MSSM against the backdrop of a highly reduced MSH pool (1 mM). Lastly, uncoupled roGFP2 remained completely non-responsive to changes in MSH/MSSM ratios (Figure 2F). Together, our results show that Mrx1-roGFP2 is exceptionally sensitive to measure physiological and dynamic changes in MSH/MSSM redox state.

### Mrx1-roGFP2 senses mycothiol redox state in mycobacteria

To investigate the redox responsiveness of Mrx1-roGFP2 in mycobacteria, we stably expressed Mrx1-roGFP2 in *Msm*. Next, we confirmed that the biosensor responds ratiometrically upon exposure of *Msm* to diamide or dithiothreitol (DTT) (Figure 2G). To examine if Mrx1-roGFP2 senses *E_MSH_* in *vivo*, we pharmacologically and genetically perturbed MSH levels in *Msm*. We first analyzed the response of Mrx1-roGFP2 upon depletion of the cytosolic MSH pool in *Msm* using dequalinium [an established small-molecule inhibitor of MSH ligase (MshC) [28]]. For comparison, we also tested inhibitors of TRX reductase (cisplatin) [29] and dihydrolipoamide dehydrogenase (5-methoxyindole-2-carboxylic acid) [30], both of which do not affect the cellular MSH pool. As expected, only dequalinium treatment led to a substantial increase in the fluorescence excitation ratio of Mrx1-roGFP2 (Figure 2H).

We further validated the MSH-specific response of Mrx1-roGFP2 by expressing it in MSH-negative (*MsmΔmshA*) and MSH-depleted (*MsmΔmshD*) strains of *Msm* [31], [32]. In addition to MSH, mycobacteria express the NADPH-dependent TRX system to efficiently counter oxidative stress [33]. Importantly, multiple components of the TRX system were down-regulated in mycobacterial strains lacking extracytoplasmic sigma factor, SigH [33], [34]. Therefore, to rule out the interaction of Mrx1-roGFP2 with the TRX system inside mycobacteria, we analyzed the biosensor response in SigH-deleted strain of *Msm* (*MsmΔsigH*). Oxidation of the biosensor was nearly quantitative (∼95%±3 oxidized) in *MsmΔmshA* as compared to ∼20%±2 in both wt *Msm* and *MsmΔsigH*, and ∼50%±4 in *MsmΔmshD* (Figure 2I). Since *MsmΔmshD* contains only 1% to 3% of total cellular MSH but produces two related thiols (Suc-mycothiol and formyl-mycothiol) [35], our data suggest that Mrx-1 can facilitate roGFP2 reduction with Suc-MSH and/or formyl-MSH, albeit suboptimally. A previous study reported a marked accumulation of ERG in *MsmΔmshA* [36]. Therefore, we also examined if Mrx1-roGFP2 can function as a sensor of ERG in mycobacteria. However, Mrx1-roGFP2 did not respond to ERG *in vitro*, further supporting mycothiol-specific response of Mrx1-roGFP2 (Figure S2A). Taken together, data generated from several independent techniques demonstrate that Mrx1-roGFP2 responds to the MSH redox buffer in mycobacteria.

Specific equilibration of Mrx1-roGFP2 with the MSH redox buffer enables precise measurement of the *E_MSH_* in various strains of *Msm* using the Nernst equation as described in SI *Materials and Methods*. Our studies reveal *E_MSH_* in wt *Msm*, *MsmΔmshA*, *MsmΔmshD*, and *MsmΔsigH* to be −300±2 mV, −239±7 mV, −275±7 mV, and −300±3 mV, respectively. Notably, the oxidizing *E_MSH_* values observed for *MsmΔmshA* and *MsmΔmshD* are consistent with our earlier findings indicating that the thiol-disulfide redox switch in Mrx1-roGFP2 is a specific substrate for the MSH reductive pathway.

### Mrx1 enhances sensitivity of roGFP2

**Figure 3:**
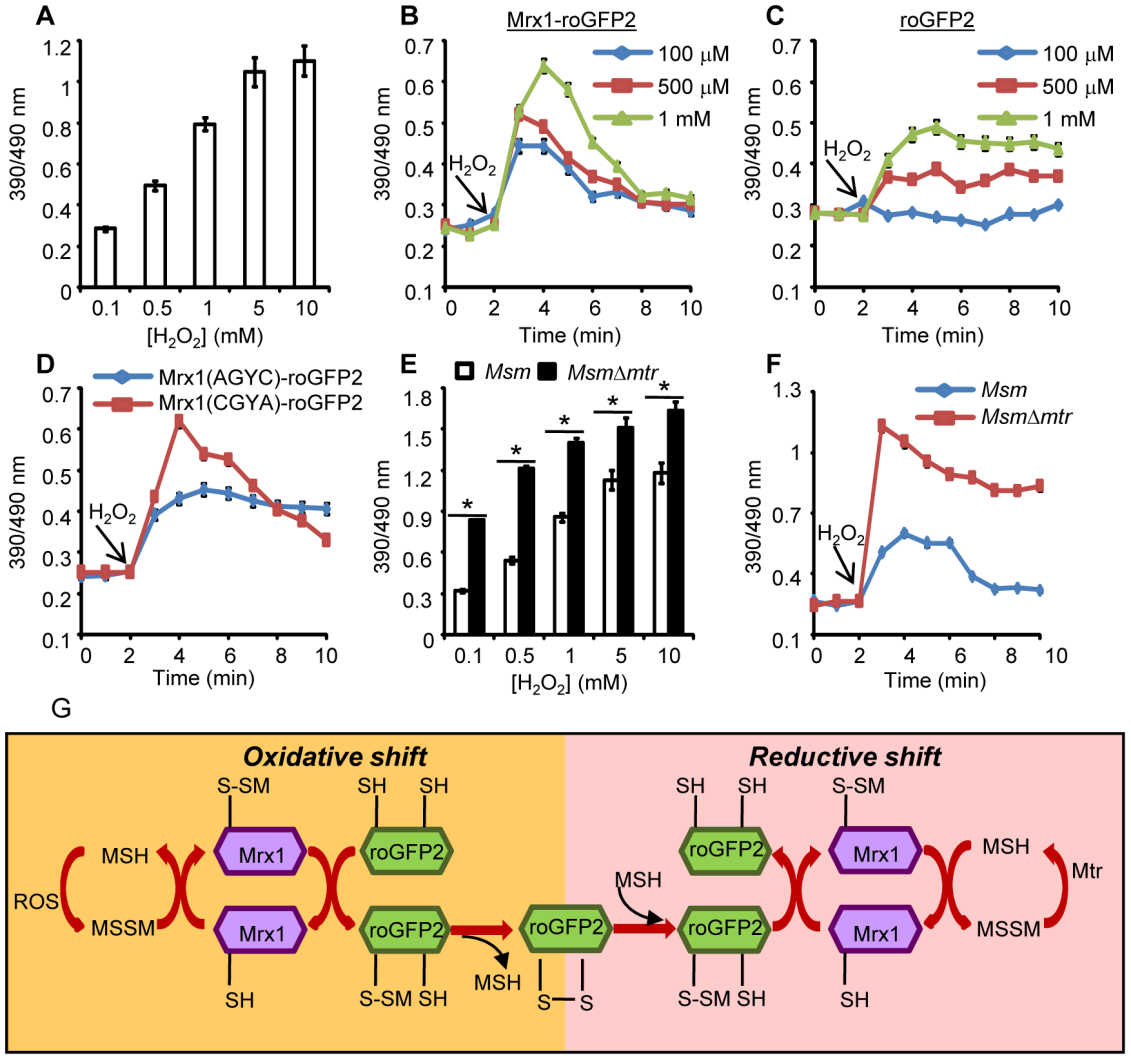
Coupling of Mrx1 with roGFP2 facilitates detection of transient redox changes in mycobacteria. (A) *Msm* expressing Mrx1-roGFP2 was treated with varying concentrations of H_2_O_2_ for 2 min and ratiometric sensor response was measured. *Msm* expressing (B) Mrx1-roGFP2 or (C) roGFP2 was treated with the indicated amounts of H_2_O_2_ and the ratiometric sensor response was measured. (D) Oxidation of *Msm* expressing Mrx1(AGYC)-roGFP2 and Mrx1(CGYA)-roGFP2 exposed to 1 mM H_2_O_2_. (E) *Msm* and *MsmΔmtr* strains expressing Mrx1-roGFP2 were oxidized with indicated concentrations of H_2_O_2_ and the ratiometric sensor response was measured. (F) *Msm* and *MsmΔmtr* strains expressing Mrx1-roGFP2 were exposed to 1 mM H_2_O_2_ and ratiometric sensor response was measured as a function of time. Error bars represent standard deviations from the mean. * p<0.01. Data are representative of at least three independent experiments. (G) Proposed molecular mechanism of the Mrx1-roGFP2 biosensor. In response to oxidative stress, the nucleophillic cysteine (C14) of Mrx1 specifically reacts with MSSM to generate a mixed Mrx1-MSSM intermediate. The Mrx1-MSSM interacts with one of the two proximal Cys thiols on roGFP2 and converts it into S-mycothionylated roGFP2. The generation of S-mycothionylated roGFP2 intermediate is subject to future experimentation. In the final step, S-mycothionylated roGFP2 rearranges to form intermolecular disulfide bond between Cys147-Cys204. This results in an oxidative shift in *E_MSH_*. Once oxidative stress depletes, *E_MSH_* normalizes predominantly via reduction of MSSM to MSH by Mtr.

We next investigated whether coupling of roGFP2 with Mrx1 enhanced the sensitivity of the new biosensor towards transient changes in intracellular *E_MSH_*. Initial studies confirmed that H_2_O_2_ alone (0.5–5 mM) does not directly oxidize the Mrx1-roGFP2 protein *in vitro* (Figure S2B). By contrast, application of H_2_O_2_ to cells led to rapid (∼2 min) and substantial oxidation of the biosensor within *Msm* (Figure 3A). These results suggest that H_2_O_2_–mediated oxidation of MSH to MSSM is necessary for biosensor oxidation in *Msm*. Also, Mrx1-roGFP2 ratio rapidly increased upon exposure of *Msm* to diverse oxidants such as menadione, aldrithiol, and diamide (Figure S2C).

The sensitivity of Mrx1-roGFP2 in *Msm* was further investigated upon exposure to lower concentrations of H_2_O_2_. Addition of 100 µM, 500 μM and 1 mM of H_2_O_2_ resulted in rapid, but short-lived (∼5 min) increases in Mrx1-roGFP2 ratio, suggesting efficient mobilization of anti-oxidant response mechanisms in *Msm* (Figure 3B). In contrast, *Msm* expressing either uncoupled roGFP2 (Figure 3C) or Mrx1(AGYC)-roGFP2 (Figure 3D) responded slowly to lower concentrations of H_2_O_2_ and did not display an anti-oxidative response. The poor response shown by uncoupled roGFP2 and Mrx1(AGYC)-roGFP2 could either be due to their non-specific interaction with other mycobacterial redox systems (*e.g.* TRX, ERG *etc*) or suboptimal equilibration with the MSH/MSSM couple through mediation by endogenous *Msm* Mrx1. Finally, Mrx1(CGYA)-roGFP2 retained transient responses similar to Mrx1-roGFP2 (Figure 3D), further substantiating the crucial role of N-terminal Cys residue of Mrx1 in promoting a rapid and reversible equilibration of biosensor with the intracellular MSH/MSSM redox buffer. To decisively show that Mrx1-roGFP2 is capable of detecting dynamic changes in *E_MSH_*, we exploited another *Msm* mutant lacking MSH disulfide reductase (Mtr) activity (*MsmΔmtr*). The Mtr enzyme maintains intramycobacterial MSSM/MSH ratios by reducing MSSM to MSH upon oxidative stress, consequently its absence results in the depletion of MSH [37]. Exposure to H_2_O_2_ led to a ∼2-fold increase in the ratio of oxidized Mrx1-roGFP2 in *MsmΔmtr* as compared to wt *Msm* (Figure 3E). Importantly, the short-lived oxidative deflections of *E_MSH_* in response to limiting amounts of H_2_O_2_ were significantly extended in the *MsmΔmtr* mutant, thereby implicating Mtr in orchestrating an efficient anti-oxidative response in *Msm* (Figure 3F). On this basis, we propose a biochemical mechanism of sensing mycobacterial *E_MSH_* using Mrx1-roGFP2 bioprobe (Figure 3G).

It can be argued that the overexpression of a redox-based enzyme in our biosensor can influence cytoplasmic MSH/MSSM redox state to compromise redox measurements. However, we found that the absolute concentration of Mrx1-roGFP2 inside *Msm* cells is 1000–10000 fold lower (1 µM/cell; Figure S2D) as compared to the high millimolar concentrations of mycothiol (1–10 mM) present in mycobacteria. This shows that reducing equivalents (thiols) introduced by the expression of biosensor are significantly less than the combined pool of MSH and other thiols present in mycobacteria. Furthermore, ambient *E*_MSH_ of *Msm* cells overexpressing either Mrx1-roGFP2 or its catalytically inactive derivative [Mrx1(AGYC)-roGFP2] was found to be comparable i. e −300±2 mV and −298±3 mV, respectively, indicating that Mrx1 activity does not perturb steady state *E*_MSH_. Lastly, Mrx1-roGFP2 harboring *Msm*, *MsmΔmshA*, and *MsmΔmtr* strains showed similar survival profile upon exposure to H_2_O_2_ as compared to control strains, suggesting no adverse influence of intracellular levels of biosensor on resistance to oxidative stress (Figure S2E). Taken together, we demonstrate that by integrating Mrx1 with roGFP2, the biosensor becomes catalytically self-sufficient in establishing a rapid and specific equilibration with the MSH redox buffer.

### Measuring *E_MSH_* of slow growing *Mtb* strains

To obtain information about the basal redox potential differences between various species and strains of mycobacteria, we expressed Mrx1-roGFP2 in vaccine strain (*M. bovis* BCG), virulent laboratory strain (*Mtb* H37Rv), and several Indian clinical isolates of *Mtb* including single-drug resistant (BND 320), multi-drug resistant (MDR - Jal 2261, 1934, Jal 2287), and extensively-drug resistant (XDR - MYC 431). First, we confirmed that overexpression of Mrx1-roGFP2 does not affect metabolic activity and growth of *Mtb* using metabolic indicator dye, Alamar blue and by measuring culture absorbance (Figure S3A and S3B). Any downstream imaging analysis of the BSL3 class pathogens requires their chemical fixation by paraformaldehyde (PFA), which we found to oxidize the biosensor (Figure S3C). To circumvent PFA-mediated oxidation artifacts, we alkylated the thiols of Mrx1-roGFP2 using the cell permeable fast-acting thiol-modifier, N-ethyl maleimide (NEM). Control experiments clearly show that NEM treatment efficiently prevents oxidation during PFA-fixation of *Mtb* cells (Figure S3C). A similar chemical-fixation strategy was successfully exploited to measure the redox potential of glutathione (*E_GSH_*) in HeLa cells [24], and in the sub-cellular compartments and tissues of Drosophila using Grx1-roGFP2 [38]. With this system in hand, we confirmed that Mrx1-roGFP2 responds ratiometrically to oxidant (cumene hydroperoxide; CHP) and reductant (DTT) in *Mtb* H37Rv (Figure S4A). A concentration and time-dependent oxidation of Mrx1-roGFP2 upon H_2_O_2_ exposure was also detected in *Mtb* H37Rv (Figure S4B and S4C). Of note, induction of anti-oxidative response upon exposure to H_2_O_2_ was significantly delayed in *Mtb* (∼120 min) as compared to rapid response observed earlier in *Msm* (∼5 min) (Figure S4C), suggesting important variations in sensing and responding to redox stress between the two species.

Next, we evaluated the redox potential of various slow growing lab-adapted and clinical mycobacterial strains. The resulting data indicates that there is relatively little variation in the redox state within and between drug-resistant clinical (MDR/XDR) and drug-sensitive lab (*Mtb* H37Rv, *M bovis* BCG) strains, as exemplified by *E_MSH_* values around −273 mV to −280 mV (Table S1). This finding suggests that the steady-state *E_MSH_* is relatively unaffected by either genotypic or phenotypic variations within *Mtb* strains under laboratory growth conditions. However, *E_MSH_* in slow growing mycobacterial strains is notably oxidizing compared to *Msm* (−300±2 mV), which is consistent with an earlier report showing higher MSH/MSSM ratio in *Msm* (200∶1) as compared to BCG (50∶1) [39]. Lastly, to rule out any contribution of the chemical fixation procedure to the observed variation in *E_MSH_* between *Mtb* and *Msm*, we treated *Msm* expressing Mrx1-roGFP2 with NEM-PFA and confirmed that *Msm* maintains *E_MSH_* of −300±2 mV.

### Macrophage induces redox heterogeneity within *Mtb* populations

**Figure 4:**
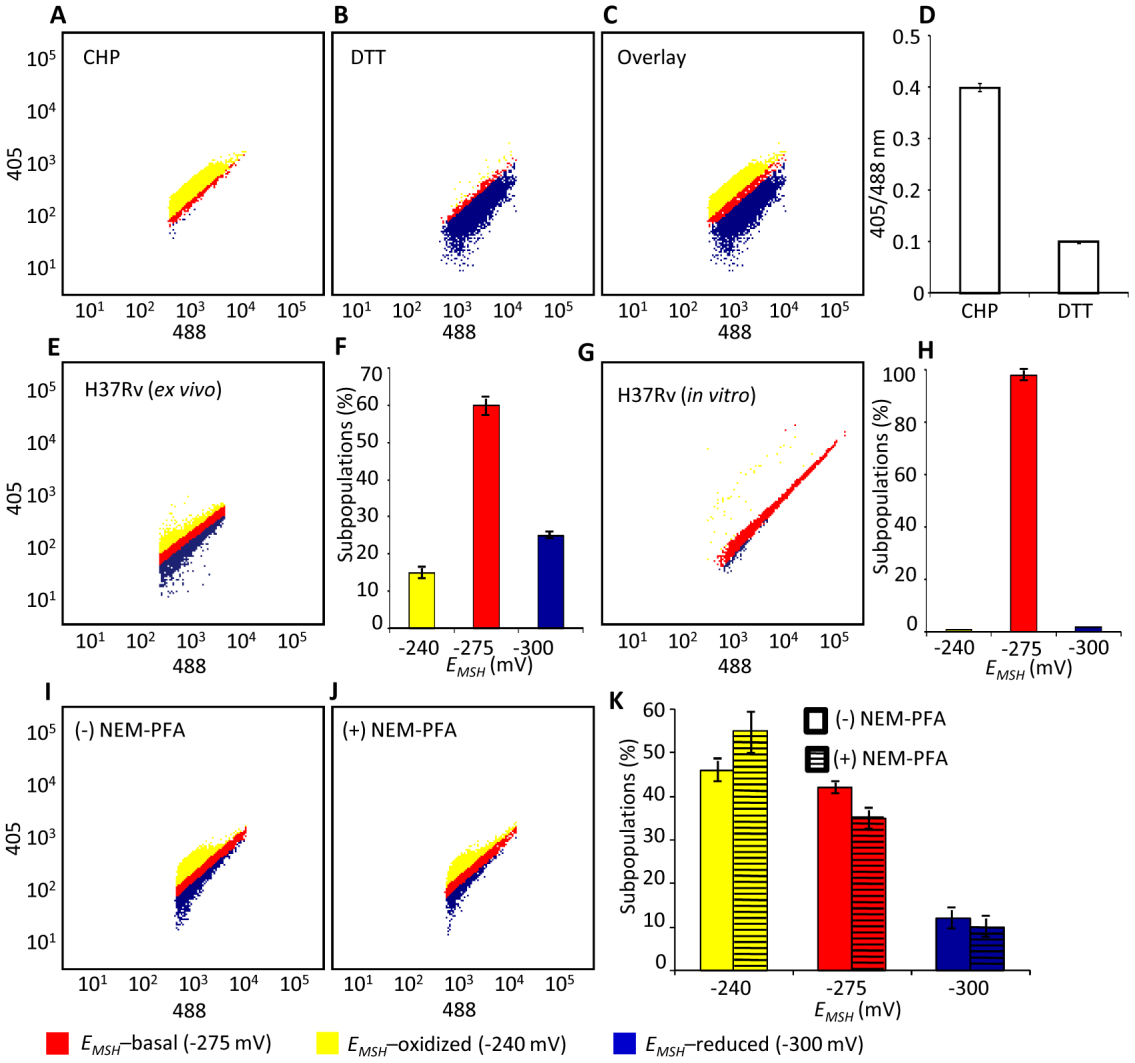
Emergence of redox heterogeneity within *Mtb* population inside macrophages. PMA-differentiated THP-1 cells were infected with *Mtb* H37Rv expressing Mrx1-roGFP2 (moi: 10) and ∼30,000 cells were analyzed by flow cytometry by exciting at 405 and 488 nm lasers at a constant emission (510 nm). The program BD FACS suite software was used to analyze the population distribution of *Mtb*, and each population was represented by an unique color. Using automatic and manual gating options, a strategy was adopted to categorize *Mtb* population into three subpopulations: *E_MSH_*-oxidized, *E_MSH_*-reduced and *E_MSH_*-basal. Number of events per subpopulation was counted and representative percentage of each subpopulation was estimated. Gates were selected on the basis of complete oxidation by 1 mM CHP (*E_MSH_*-oxidized) and complete reduction by 10 mM DTT (*E_MSH_*-reduced). Dot plots show shift in population towards oxidizing or reducing after treatment with (A) CHP and (B) DTT, respectively. (C) Overlay spectra of dot plots derived from CHP and DTT treatment of infected THP-1 cells. (D) Bar graph represents the ratiometric sensor response. (E) Dot plot of THP-1 cells infected with H37Rv expressing Mrx1-roGFP2 at 72 h p.i. (F) Bar graph represents percentage of bacilli in each subpopulation. (G) Dot plot of H37Rv expressing Mrx1-roGFP2 grown in 7H9 medium. (H) Bar graph represents percentage of bacilli in each subpopulation. (I) THP-1 cells were infected with BCG expressing Mrx1-roGFP2 and ∼30,000 cells were analyzed by flow cytometry at 24 h p.i. (J) In a parallel set, BCG infected cells were first fixed by NEM and PFA followed by flow cytometry. (K) Shown is the dot plot of BCG infected THP-1 cells with and without NEM-PFA treatment. Note that redox heterogeneity was preserved independent of NEM-PFA treatment. Bar graph represents percentage of bacilli in each subpopulation. The *E_MSH_* of mycobacterial cells *in vitro* and inside macrophages was calculated by fitting Mrx1-roGFP2 ratios into the *in vitro* redox calibration curve (see SI *Materials and Methods*). Color codes representing each subpopulation with a defined average *E_MSH_* in the panels are shown at the bottom of the figure. Error bars represent standard deviations from the mean of at least three independent experiments.

We next determined whether Mrx1-roGFP2 could be used to quantify redox changes that occur in the natural context of infection. To investigate this issue, we infected THP-1 macrophages with *Mtb* H37Rv expressing Mrx1-roGFP2 at a multiplicity of infection (moi) of 10 and monitored intramycobacterial *E_MSH_*. To do this, we performed NEM-PFA based fixation technique followed by ratiometric fluorescence analysis by flow cytometry (see SI *Materials and Methods*). Since the flow cytometric based measurements are dependent on fixed wavelength lasers, we excited Mrx1-roGFP2 biosensor with the canonical 405 and 488 nm laser wavelengths at a fixed emission wavelength of 510 nm (see SI *Materials and Methods*). We first confirmed that intramycobacterial Mrx1-roGFP2 responds ratiometrically to oxidant; CHP and reductant; DTT inside macrophages (Figure 4A, 4B, 4C, and 4D). To measure changes in *E_MSH_* during infection, an *in vitro* redox calibration curve was generated by treating *Mtb* H37Rv with buffers of known redox potentials. By fitting Mrx1-roGFP2 ratio to the redox calibration curve, we precisely calculated the *E_MSH_* of *Mtb* inside macrophages (Figure S5A, see SI *Materials and Methods*). Intriguingly, flow cytometric analyses of ∼30,000 infected macrophages demonstrated the presence of cells with a gradient of intramycobacterial *E_MSH_*. For the purpose of measurements, infected macrophages were gated into three subpopulations on the basis of their corresponding intramycobacterial *E_MSH_* (Figure 4E and 4F). An *E_MSH_*-basal population with an intermediate *E_MSH_* of −275±5 mV, and two deflected populations were observed (Figure 4E and 4F). Deflected cells with a mean *E_MSH_* of −240±3 mV represent an *E_MSH_*-oxidized subpopulation, based on the observation that CHP treatment of infected macrophages results in a significant fraction of these gated cells (∼98%) (Figure 4E and 4F). The population with an average *E_MSH_* of −300±6 mV represents an *E_MSH_*-reduced subpopulation, as treatment of infected macrophages with the DTT results in ∼96% of the cells gating into this subpopulation (Figure 4E and 4F). *Mtb* cells present in media alone and analyzed in parallel did not show redox heterogeneity (Figure 4G and 4H), suggesting that the intramacrophage environment perturbs redox homeostasis to induce redox variability in *Mtb*. Furthermore, we infected macrophages with BCG, and measured intramycobacterial *E_MSH_* with and without NEM-PFA treatment. Both conditions induce similar degree of heterogeneity in intramycobacterial *E_MSH_* (Figure 4I, 4J, and 4K), demonstrating that redox heterogeneity has a biological basis and is not due to aberrant quenching of fluorescent signals during NEM-PFA treatment.

**Figure 5:**
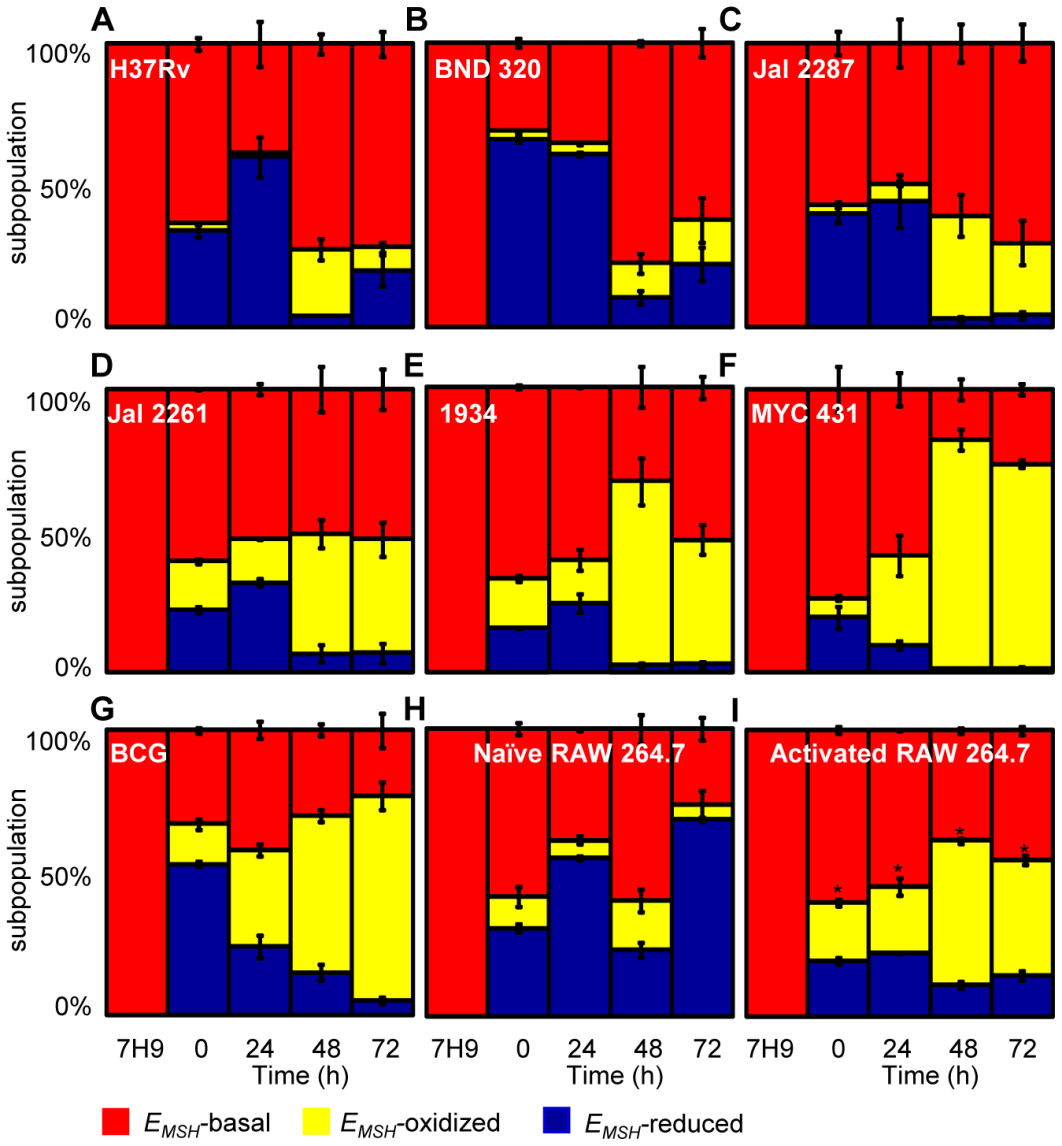
Dynamic changes in intrabacterial *E_MSH_* during infection. THP-1 cells were infected with Mrx1-roGFP2 expressing *Mtb* strains; (A) H37Rv, (B) BND 320, (C) Jal 2287, (D) 1934, (E) Jal 2261 (F) MYC 431 and (G) BCG at an moi of 10. (H) Naïve and (I) IFN-γ/LPS treated (activated) RAW 264.7 macrophages were infected with H37Rv (moi: 10). At indicated time points, cells were treated with NEM-PFA and ∼30,000 infected macrophages were analyzed by flow cytometry and intramycobacterial *E_MSH_* was measured as described in figure 4. The “0” h time point refers to time immediately after initial infection with H37Rv for 4 h. The percentage of bacilli in each subpopulation was calculated and plotted as a bar graph. Data shown is the result of at least six independent experiments performed in quadruplicate and data from biologically independent experiments were combined and represented as percentage of bacilli in each subpopulation ± standard deviation. p-values shown in the panel I were calculated by comparing *E_MSH_*-oxidized populations of resting and activated macrophages (* p<0.01).

With the flow cytometry workflow in hand, we measured time-resolved changes in intramycobacterial *E_MSH_* during infection of THP-1 macrophages. Our results indicated that the initial period (0–24 h post-infection [p.i.]) of infection was associated with a gradual increase in cells with reduced *E_MSH_* (60±7%) followed by an oxidative shift (25±5%) at 48 h p.i. and then a significant recovery from oxidative stress, as revealed by a decrease in the population with oxidized *E_MSH_* (7±3%) at 72 h p.i. (Figure 5A). The observed redox heterogeneity and oscillatory patterns were confirmed by repeating experiments at least six times in quadruplicate and data from biologically independent experiments were combined and presented as mean ± standard deviation. We also verified the presence of both time-dependent heterogeneity and oscillations in intramycobacterial *E_MSH_* upon infection of THP-1 macrophages at a low moi of 1 (Figure S5B). However, we noticed that infection with lower moi induced higher proportion of bacteria with oxidized *E_MSH_* at each time point examined as compared to cells infected at a moi of 10 (Figure S5B and S5C).

Next, we investigated if the macrophage environment induces strain-specific variations in redox heterogeneity among various slow growing mycobacteria including BCG and Indian clinical MDR/XDR isolates. For this, we infected THP-1 macrophages with various strains of *Mtb* at a moi of 10 and time-resolved changes in intramycobacterial *E_MSH_* were measured as described in the earlier section. Interestingly, while heterogeneity in *E_MSH_* for BND 320 largely followed the reductive-oxidative-reductive oscillatory pattern of H37Rv (Figure 5B), distinct redox deviations were displayed by other strains. For example, Jal 2287, Jal 2261 and 1934 displayed overrepresentation of the *E_MSH_*-oxidized subpopulation at 48 h p.i. followed by a poor recovery at 48–72 h p.i., as compared to H37Rv (Figure 5C–5E). Noticeably, intramacrophage growth of MYC 431 displayed a loss of redox oscillatory pattern and showed a steady increase in *E_MSH_*-oxidized subpopulation over 48 h p.i. (Figure 5F). Intriguingly, intramacrophage profile of BCG displayed temporal changes in *E_MSH_* comparable to Jal 2261, 1934, and MYC 431. As shown in Figure 5G, infection with BCG showed a continuous decrease in *E_MSH_*-reduced subpopulation with a concomitant increase in *E_MSH_*-oxidized subpopulation over time (Figure 5G). Together, these findings for the first time revealed that macrophage environment triggers heterogeneity in *E_MSH_* of *Mtb* and uncovered redox variance among clinical field isolates.

### Immune activation of macrophages induces oxidative shift in *E_MSH_* of *Mtb*

In order to examine the biosensor response upon stimulation of oxidant-mediated antimycobacterial stresses, we performed additional experiments in immunologically activated murine macrophages (RAW 264.7). Activated murine macrophages are known to control mycobacterial proliferation by producing ROS and RNS [2]. RAW 264.7 were activated with IFN-γ and LPS prior to infection with *Mtb* H37Rv [11]. Ratiometric flow cytomteric analysis showed a significant and sustained oxidative shift in *E_MSH_* of *Mtb* H37Rv inside activated macrophages at each time point investigated, whereas intramycobacterial *E*_MSH_ inside naïve macrophages showed redox oscillations similar to THP-1 cells (Figure 5H and 5I). These results indicate that *Mtb* cells were able to recover from mild oxidative stress conditions inside naïve macrophages, whereas recovery was compromised in IFN-γ/LPS primed macrophages. Since nitric oxide (NO) generated via iNOS is considered to be one of the major contributors of redox stress in *Mtb* inside immune-activated murine macrophages [40], we treated IFN-γ/LPS activated RAW 264.7 macrophages with a well established iNOS inhibitor N^G^-methyl-L-arginine (NMLA;[41]) and monitored intramycobacterial *E_MSH_*. Strikingly, a substantial reduction in subpopulation with oxidized *E_MSH_* was observed upon addition of NMLA (Figure S6A). These results indicate that *Mtb* responds to host derived environmental cues by modulating *E_MSH_*, and further illustrates the utility of Mrx1-roGFP2 in dissecting redox signaling during infection.

### Sub-vacuolar compartments are the source of redox heterogeneity

**Figure 6:**
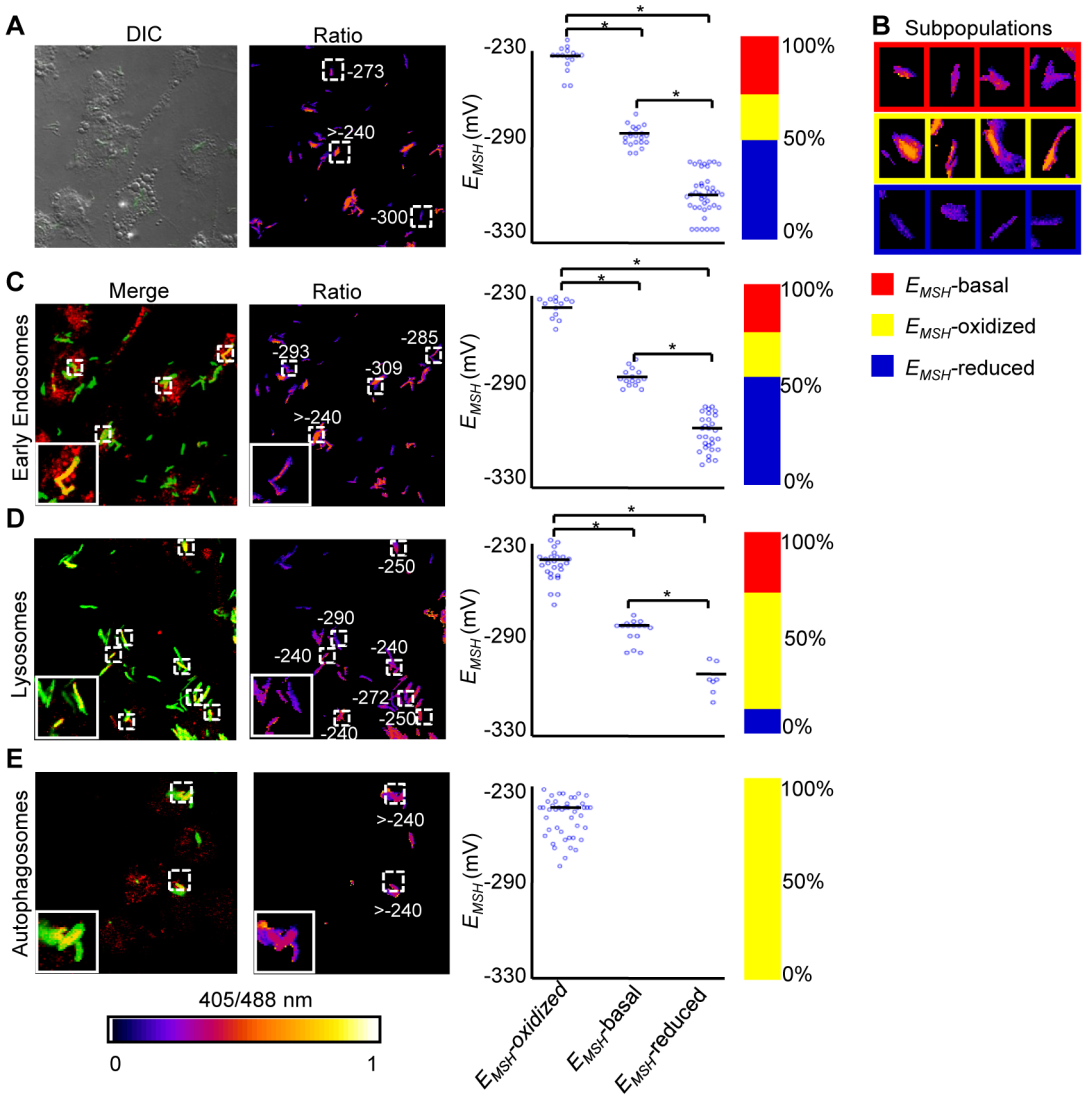
Sub-vacuolar compartments are the source of redox heterogeneity within *Mtb* population during infection. THP-1 cells were infected with H37Rv expressing Mrx1-roGFP2 (moi: 10). At 24 h p.i. infected cells were treated by NEM-PFA. Cells were then stained for EEA1 and LC3 and analyzed by confocal microscopy for measuring ratiometric sensor response in *Mtb* co-localized within early endosomes and autophagosomes, respectively. In case of lysosomes, the cells were first pre-treated with Lysotracker followed by NEM-PFA fixation. (A) False color ratio confocal image of *Mtb* (∼80) inside THP-1 at 24 h p.i. *E_MSH_* of bacilli was measured using *in vitro* calibration curve (SI Figure S5A). (B) Representative bacilli for each subpopulation are shown. Co-localization of *Mtb* H37Rv in (C) endosomes, (D) lysosomes, and (E) autophagosomes. The co-localization is demonstrated in the merged images, where green indicates bacteria, red indicates phagosomal markers, and yellow indicates a positive correlation. False color ratio images were generated as described in SI *Materials and Methods*. Small dashed line boxes indicate co-localized bacilli and large solid line boxes represent the enlarged view of the one of the co-localized bacilli. Numbers represent *E_MSH_* in millivolts. *E_MSH_* of co-localized bacilli (≥50) is calculated and distribution is shown in scatter plot. In each panel, scatter plot depicts quantification of microscopy data. Each point on the plot represents a bacterium. Bar represents mean values. p-values were calculated by one way ANOVA followed by Tukey's HSD statistical test (* p<0.01). Percentage of bacilli in each subpopulation is represented as a stacked bar graph in every panel. Color bar corresponds to the 405/488 nm ratios ranging from 0 to 1. Data shown is the representative of at least three independent experiments.

Intracellular *Mtb* exists in different vacuolar compartments, which may contribute to significant heterogeneity in mycobacterial gene expression, metabolic state and survival [42]. On this basis, we next asked whether trafficking into distinct vacuolar compartments could promote redox heterogeneity in the *Mtb* population using ratiometric confocal microscopy. First, we performed confocal imaging of *Mtb* cells inside THP-1 macrophages at 24 h p.i. Conforming to our flow cytometric findings, confocal analyses revealed that the intrabacterial *E_MSH_* varied markedly at a single-cell level. Similar to flow cytometry, this gradient in redox heterogeneity can be classified into *E_MSH_*-basal (−277±5 mV, 26%), *E_MSH_*-oxidized (−242±6 mV, 23%), and *E_MSH_*-reduced (−304±10 mV, 51%) sub-populations (Figure 6A and 6B). *Mtb* grown in media indicated an overrepresentation of the cells with uniform *E_MSH_* (Figure S6B), validating that both flow cytometry and confocal imaging revealed very similar ratiometric changes upon infection.

**Figure 7:**
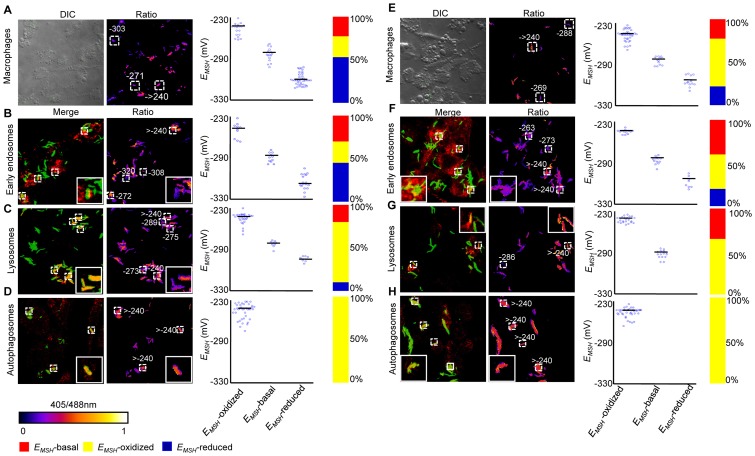
Redox heterogeneity in drug-resistant patient isolates during infection. Infection of THP-1 macrophages, confocal microscopy, and *E_MSH_* measurements were performed as described for Figure 6. False color ratio confocal image of Jal 2287 (A) and MYC 431 (E) inside THP-1 at 24 h p.i. Small dashed line boxes indicate bacilli with varying *E_MSH_*. Co-localization of *Mtb* in (B and F) endosomes, (C and G) lysosomes, and (D and H) autophagosomes. False color ratio images were generated as described in SI *Materials and Methods*. Small dashed line boxes indicate co-localized bacilli and large solid line boxes represent the enlarged view of one of the co-localized bacilli. Relative distribution of *Mtb* subpopulations with varying *E_MSH_* is depicted as scattered plots and stacked bar graphs. Each point on the plot represents a bacterium. Approximately, ≥80 individual bacilli within macrophages and ≥50 individual bacilli within each compartment were imaged to calculate *E_MSH_*. Color bar corresponds to the 405/488 nm ratios ranging from 0 to 1. Data shown is representative of at least three independent experiments.

Next, we measured intrabacterial *E_MSH_* within early endosomes, lysosomes, and autophagosomes by visualizing the co-localization of *Mtb* H37Rv expressing Mrx1-roGFP2 with compartment specific fluorescent markers at 24 h p.i. using confocal microscopy (see SI *Materials and Methods*). For marking early endosomes, cells were stained with anti- early endosome autoantigen (EEA1) and anti-Rab5 antibodies. To study lysosomes, we used acidotropic dye Lysotracker and anti-cathepsin D antibody, whereas autophagosomes were labeled with anti-LC3 antibody (see SI *Materials and Methods*). The resulting data show that the majority of bacilli within early endosomes were likely to exhibit reduced (∼54%) as compared to oxidized (∼22%) or basal (∼24%) *E_MSH_* (Figure 6C and Figure S7A). Interestingly, in lysosomes, deflected subpopulations with *E_MSH_*-oxidized were clearly higher in proportion (58%), whereas *E_MSH_*-reduced (12%) and *E_MSH_*-basal (30%) subpopulations were underrepresented (Figure 6D and Figure S7B). The percent distribution of subpopulations with *E_MSH_*-reduced and *E_MSH_*-oxidized were significantly different between early endosomes and lysosomes (p<0.001), while *E_MSH_*-basal subpopulation remained comparable within these compartments. Furthermore, 100% of the *Mtb* population inside autophagosomes displayed a maximal oxidative shift in *E_MSH_* (Figure 6E). We also measured changes in *E_MSH_* during intramacrophage residence of drug-resistant strains Jal 2287 and MYC 431. While Jal 2287 displayed redox deviations similar to *Mtb* H37Rv (Figure 7A–7D), MYC 431 showed over-representation of subpopulations with *E_MSH_*-oxidized within the macrophage and sub-vacuolar compartments at 24 h p.i. (Figure 7E–7H). Taken together, our results suggest that distinct sub-vacuolar environments lead to the generation of *Mtb* subpopulations with a gradient of redox potentials.

### Anti-TB drugs induce oxidative stress in *Mtb* during infection

**Figure 8:**
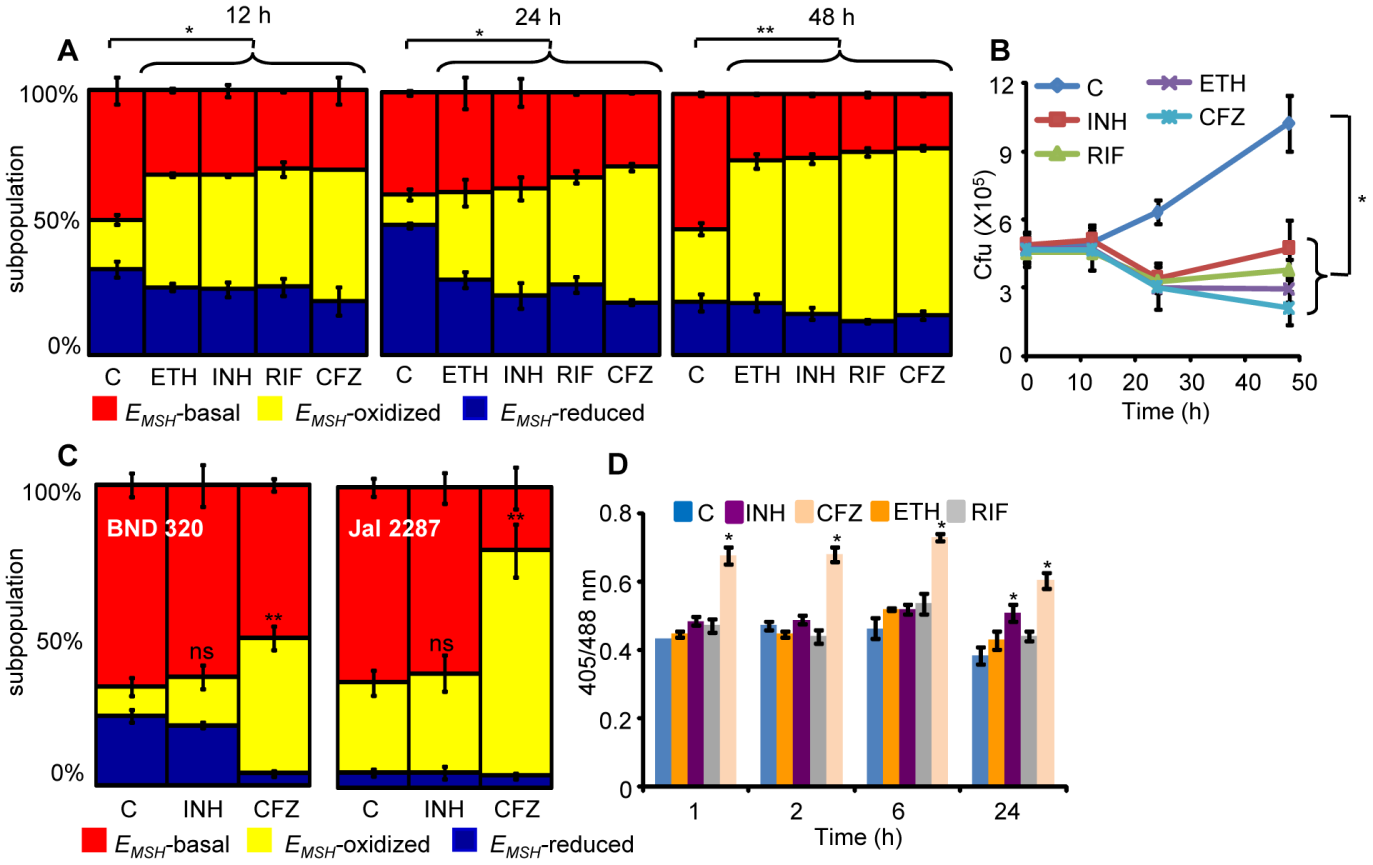
Anti-TB drugs induce oxidative shift in *E_MSH_* of *Mtb* during infection. THP-1 cells infected with *Mtb* H37Rv expressing Mrx1-roGFP2 were treated with ETH (5 µg/ml), INH (0.5 µg/ml), RIF (0.5 µg/ml) and CFZ (0.5 µg/ml) immediately after infection. (A) At indicated time points redox heterogeneity within *Mtb* cells was analyzed by flow cytometry. (B) In a parallel experiment, infected macrophages were lysed and released bacilli were counted by plating for CFU. (C) THP-1 cells infected with the clinical strains BND 320 (INH mono-resistant) and Jal 2287 (MDR) were exposed to INH (0.5 µg/ml) and CFZ (0.5 µg/ml) immediately after infection and redox heterogeneity within *Mtb* cells 48 h p.i. was analyzed by flow cytometry. p-value shown in each panel was calculated by comparing untreated (control) and anti-TB drugs treated groups. (D) *Mtb* H37Rv expressing Mrx1-roGFP2 grown till exponential phase in 7H9-ADS medium was independently treated with ETH (5 µg/ml), INH (0.5 µg/ml), RIF (0.5 µg/ml) and CFZ (0.5 µg/ml) and ratiometric sensor response was measured at indicated time points. C: represents untreated control at each time point. Error bars represent standard deviations from the mean. * p<0.01, **p<0.001, ns: not significant. Data are representative of at least three independent experiments.

Several studies have shown that MSH-deficient mycobacteria and other actinomycetes are sensitive to antibiotics [21], [43], [44]. On the other hand, MSH also contributes to susceptibility to INH and ETH [45], and mutations in mycothiol biosynthesis genes were identified in drug-resistant clinical isolates of *Mtb* [46]. While these constitute an important foundation linking antibiotic action with mycothiol redox homeostasis *in vitro*, they provide little insight into how antibiotics modulate intramycobacterial *E_MSH_* in the physiological setting of infection. To examine this, we characterized the effect of anti-TB drugs on redox heterogeneity in *Mtb* cells during intramacrophage residence. To this end, infected macrophages were exposed to anti-TB drugs (5-fold the *in vitro* MIC) with different modes of action (*e.g.* isoniazid [INH; mycolic acid inhibition], ethambutol [EMB; arabinogalactan inhibition], rifampicin [RIF; inhibition of transcription], and clofazimine [CFZ; redox cycling and ROS production; [47]) and the redox response was measured by flow cytometry. Treatment with all antibiotics induced variable levels of oxidative shift in *E_MSH_* of *Mtb* subpopulations at 12, 24, and 48 h p.i. (Figure 8A). The skew towards oxidizing *E_MSH_* was activated at an early time point (12 h p.i.) and increased significantly at 48 h p.i. (Figure 8A). Next, we examined if enhanced oxidizing *E_MSH_* correlated with the killing potential of anti-TB drugs during infection. At 12 h post-antibiotic treatment, the *Mtb* survival rate was comparable to the untreated control, as determined by colony forming unit (CFU) assay (Figure 8B). However, a modest (∼1.5-fold) to a significant reduction (∼5-fold) in intramacrophage bacillary load as compared to untreated control was detected at 24 and 48 h p.i., respectively (Figure 8B). These findings show that bactericidal antibiotics with different mechanisms of action induce oxidative changes in intramycobacterial *E_MSH_* during infection. To further validate these results, we infected macrophages with INH resistant clinical strains (BND 320 and Jal 2287) and measured intramycobacterial *E_MSH_* in response to INH at 48 h p.i. Since these strains are sensitive to CFZ, we used CFZ as a positive control in this experiment. Figure 8C clearly shows that the *E_MSH_* of strains genetically resistant to INH remained uninfluenced in response to INH. On the other hand, CFZ exposure generated considerable oxidative shift in *E_MSH_* of these strains (Figure 8C).

Lastly, to investigate if anti-TB drugs directly induce oxidative stress *in vitro*, we exposed *Mtb* cells grown in 7H9 medium supplemented with albumin, dextrose and sodium chloride (7H9-ADS) to INH, CFZ, RIF, and ETH (5× MIC) and tracked *E_MSH_* at 1, 2, 6, and 24 h post-treatment. This study revealed that only CFZ induces significant oxidative shift in *E_MSH_* of *Mtb* (Figure 8D). Other anti-TB drugs such as INH induce low levels of *E_MSH_*-oxidized at 24 h, post-treatment, while RIF and ETH do not influence the *E_MSH_* of *Mtb* (Figure 8D). Our results suggest that antibiotics do not perturb *E_MSH_* of *Mtb per se*, but co-opt host cellular responses to stimulate excessive oxidative stress during infection. These findings underscore the importance of studying redox-based mechanisms of drugs action under physiologically relevant microenvironmental conditions such as those encountered during TB infection in macrophage.

### Redox heterogeneity induces differential susceptibility to anti-TB drugs

**Figure 9:**
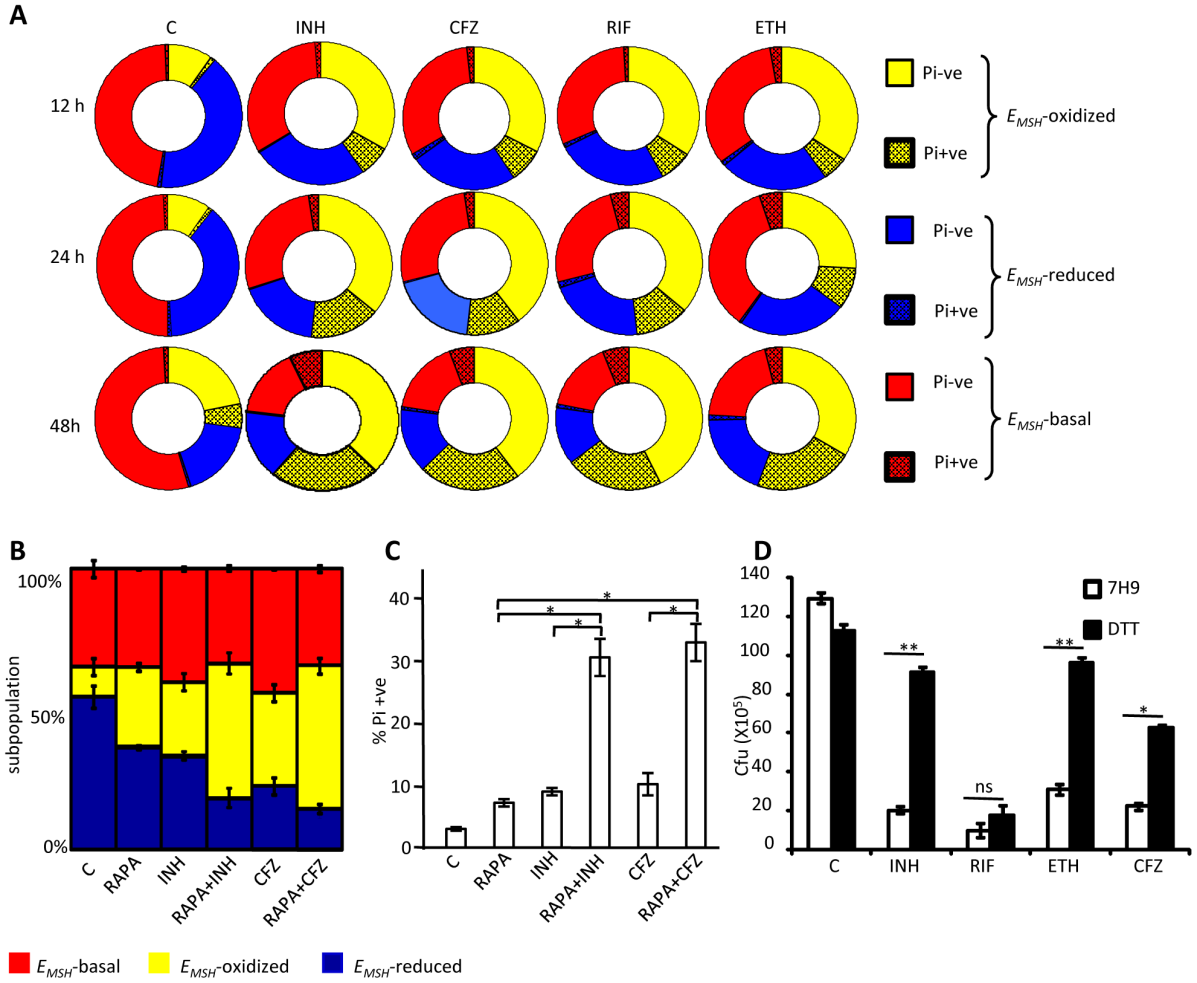
Heterogeneity in intrabacterial *E_MSH_* modulates drug tolerance. (A) THP-1 cells infected with *Mtb* H37Rv were treated with INH (0.5 µg/ml), CFZ (0.5 µg/ml), RIF (0.5 µg/ml) and ETH (5 µg/ml). At indicated time points, intracellular bacteria were fixed with NEM, released from macrophages, and stained with Pi. Pi status and *E_MSH_* of bacterial cells were determined using multi-parameter flow cytometric analysis. Pie charts display the percentage of Pi+ve and Pi-ve cells in each subpopulation. (B) THP-1 cells infected with *Mtb* H37Rv were treated with INH (0.5 µg/ml), CFZ (0.5 µg/ml), with or without rapamycin (200 nM) immediately after infection. At 24 h p.i. redox heterogeneity within *Mtb* cells was analyzed by flow cytometry. (C) In a parallel experiment, infected macrophages were lysed and released bacilli were stained with Pi. (D) Exponentially grown culture of *Mtb* H37Rv was treated with INH (0.5 µg/ml), CFZ (0.5 µg/ml), RIF (0.5 µg/ml) and ETH (5 µg/ml) in the presence or absence of 5 mM DTT (added at 0 and 2^nd^ day, post-antibiotic treatment) and number of bacilli were counted by plating for CFU. Error bars represent standard deviations from the mean. C: represents untreated control in each panel. Error bars represent standard deviations from the mean. * p<0.05, ** p<0.001, ns: not significant. Data are representative of at least three independent experiments.

It has been suggested that long term anti-TB therapy is required because the mycobacterial population is functionally heterogeneous and harbors cells that are differentially sensitive to antibiotics [48]. However, the physiological determinants of phenotypic heterogeneity in *Mtb* population and its relation with antibiotic tolerance remains poorly characterized. Because macrophage environment quickly creates variability in mycobacterial cells to generate drug tolerant subpopulations [49], we hypothesize that heterogeneity in intrabacterial *E_MSH_* may be one of the factors that underlies emergence of *Mtb* populations with differential antibiotic susceptibility. We therefore sought to determine the susceptibility of *Mtb* cells with basal, oxidized, and reduced *E_MSH_* to antibiotics during infection of THP-1 cells. To do this, we analyzed the membrane integrity of *Mtb* cells expressing biosensor by assessing their capacity to exclude fluorescent nucleic-acid binding dye, propidium iodide (Pi), upon treatment with antibiotics during infection. The state of the bacterial membrane is a crucial physiological indicator, as Pi^+^ cells are considered damaged or dying [50]. Importantly, we found that NEM treatment of infected macrophages fixed the redox state of intracellular *Mtb* such that bacterial cells released from macrophages retained redox variations comparable to bacteria within macrophages (Figure S8). This allowed us to quantify bacterial viability by Pi staining of *Mtb* cells released from infected macrophages at various time points post antibiotic exposure. Infected THP-1 cells were exposed to anti-TB drugs (5× MIC) and intracellular bacteria were fixed with NEM at 12, 24, and 48 h p.i. Infected macrophages were lysed; released bacteria were stained with Pi, and ∼30,000 bacilli were analyzed by multi-parameter flow cytometry to simultaneously profile *E_MSH_* and viability status. As shown earlier, all antibiotics induce significant oxidative shift in intramycobacterial *E_MSH_* during infection. Furthermore, bacilli with oxidized *E_MSH_* were more sensitive to killing as evident by a time-dependent increase in Pi staining across all antibiotic treatments within this subpopulation as compared to other two subpopulations (Figure 9A, p<0.05). At 48 h p.i., 35–40% of *E_MSH_*-oxidized bacilli were Pi+. The *E_MSH_*-basal subpopulation demonstrates a modest increase in Pi+ staining (∼5–10%) at 24 and 48 h p.i. (Figure 9A). Surprisingly, *E_MSH_*-reduced subpopulation remained completely unaffected by antibiotics as shown by the absence of Pi+ cells within this group (Figure 9A). These results show that bacteria with lower *E_MSH_* are capable of excluding Pi and therefore maintain membrane integrity post-antibiotic treatment.

Thus, we find that redox heterogeneous bacteria vary in their susceptibility to antibiotics, consistent with the model that macrophage induced heterogeneity in intrabacterial *E_MSH_* creates physiologically distinct subpopulations of cells. Since environment inside autophagosomes induces substantial oxidative shift in intrabacterial *E_MSH_*, we hypothesized that treatment of infected macrophages with a well established autophagy inducer (rapamycin) would increase the localization of *Mtb* to autophagosomes and shift redox heterogeneity towards *E_MSH_*-oxidized. This would allow us to examine the influence of host antibacterial mechanisms (i. e autophagy) on intrabacterial *E_MSH_* and drug tolerance during infection. To examine this, infected macrophages were treated with a low non-lethal concentration of rapamycin (200 nM) and antibiotic-mediated redox stress and killing was monitored by assessing *E_MSH_* and Pi status. As shown in figure 9B, treatment of infected macrophages with rapamycin significantly increases fraction of *Mtb* cells exhibiting oxidizing *E_MSH_* as compared to untreated control (P<0.05). Furthermore exposure of infected macrophages to both rapamycin and antibiotics (INH or CFZ) induces oxidative shift in *E_MSH_* which supersedes that produced by either INH or CFZ or rapamycin alone (Figure 9B, p<0.05). Consistent with this, treatment of infected macrophages with rapamycin-INH or rapamycin-CFZ substantially increases the fraction of Pi^+^ *Mtb* cells, indicating augmented bacterial death by these combinations (Figure 9C, p<0.05).

Lastly, to show that *E_MSH_*-reduced subpopulation contributes to drug tolerance, we measured the sensitivity of *Mtb* towards anti-TB drugs in the presence of reducing agent DTT *in vitro*. First, we confirmed that exogenous addition of 5 mM DTT maintained a reductive *E_MSH_* (−320±2.9 mV) equivalent to the *E_MSH_* of reducing subpopulation and is non-deleterious for *Mtb*. In the absence of DTT treatment, antibiotics exposure led to a significant reduction (6–12-fold) in the survival of *Mtb* (Figure 9D). By contrast, ∼80% of *Mtb* survived an exposure to INH or ETH and ∼50% in the case of CFZ in the presence of DTT (Figure 9D). Collectively, our data suggest that oxidized *E_MSH_* potentiates antibiotic action, whereas *E_MSH_*-reduced promotes tolerance to anti-TB drugs and suggest that host-induced cell-to-cell variation in *E_MSH_* may be a novel mechanism by which *Mtb* resists antimicrobial treatment during infection.

## Discussion

Basic research on persistence and drug-tolerance in *Mtb* is hampered by the lack of tools to study bacterial physiology during infection. Here, we developed a new biosensor, Mrx1-roGFP2, to image dynamic changes in the *E_MSH_* of *Mtb* during infection. Using confocal and flow cytometry, we quantified *E_MSH_* and unraveled mycothiol-linked redox heterogeneity in *Mtb* at the single-cell level during infection and highlighted the utility of bioprobes in exploring new mechanisms of drug action in *Mtb* bacilli. In Mrx1-roGFP2, the genetic coupling of roGFP2 with the mycothiol-specific oxidoreductase (Mrx1) ensures that roGFP2 functions as a physiological substrate for Mrx1 and therefore dynamically oxidizes and reduces in response to *E_MSH_*. Since cytoplasmic levels of chromosomally encoded Mrx1 may differ between mycobacterial species/strains or under diverse environmental conditions, one of the main advantages of coupling Mrx1 with roGFP2 is to facilitate rapid and continuous equilibration of biosensor with MSH/MSSM redox couple independent of endogenous Mrx1 pool. Our results agree with a recent study which showed that the homologue of *Mtb* Mrx1 in *Msm* specifically interacts with the mycothiol redox system *in vitro* [25]. Related non-disruptive approaches have been employed to develop GSH-specific and H_2_O_2_-specific *in vivo* bioprobes [24], [51].

The utility of Mrx1-roGFP2 in investigating mycobacterial physiology during infection comes from our flow cytometric and confocal data showing that the environment inside macrophage induces redox heterogeneity and oscillations in intrabacterial *E_MSH_*. The reductive-oxidative-reductive oscillations in *E_MSH_* during intramacrophage growth corresponds to the early induction of genes linked to reductive stress (*e.g.*, *whiB3*, *whiB7*, and *dosR*) [10], [42], followed by extensive bacterial killing during intermediate phase of relative increase in subpopulation with oxidized *E_MSH_* at 48 h p.i as compared to 24 h p.i., and progressive increase in replication upon anti-oxidative shifts in *E_MSH_* at later stages of infection [42]. Interestingly, we observed some variations between confocal microscopy and flow cytometry based measurements of intrabacterial *E_MSH_*. For example, a higher percentage of subpopulation with oxidized *E_MSH_* was detected using confocal microscopy as compared to flow cytometry at 24 h p.i. (Figure 5A and 6A). However, although flow cytometry allows monitoring of large number of cells at multiple time points with high statistical power, the read out is derived from averaging *Mtb* cells inside infected macrophages. Therefore, confocal microscopy is a much more accurate indicator of intrabacterial *E_MSH_* at the level of individual bacteria inside macrophages. In agreement to this, the moi dependent variations in the distribution of subpopulations with different *E_MSH_* could also be due to averaging relatively higher number of bacteria present inside macrophages infected at a moi of 10 than 1 by flow cytometry. Nonetheless, both techniques were in reasonable agreement and complement one another to generate a highly resolved view of intrabacterial *E_MSH_* during infection. We also discovered that sub-vacuolar compartments such as endosomes, lysosomes and autophagosomes are the source of redox variability in *Mtb* populations. While lysosomes and autophagosomes enrich *E_MSH_*-oxidized bacteria, early endosomes induce a reductive shift in *E_MSH_* of *Mtb*. Similarly, a greater fraction of bacteria displayed oxidized *E_MSH_* at a moi of 1 than 10, which is consistent with the reported increase in phagosomal maturation and intracellular trafficking of *Mtb* to lysosomes at low moi [52]. Finally, our results showing a substantial oxidative shift in *E_MSH_* inside activated macrophages and its reversal upon treatment with iNOS inhibitor (NMLA) are in agreement with studies implicating the role of vacuolar NOX2 and iNOS systems in creating overwhelming oxidative stress within lysosomes and autolysosomes [2]. It is well known that *Mtb* actively remodels endosomal/phagosomal pathways during infection [53] to induce variability among phagosomes during infection [54]. This suggests that *Mtb*-induced alterations within sub-vacuolar compartments can generate a range of environmental conditions for the evolution of redox deviations in *Mtb* population.

Intriguingly, we found that redox heterogeneity is uniformly present in H37Rv and MDR/XDR strains inside macrophages. However, distinct strain-dependent variations in redox heterogeneity were also evident. Because macrophage compartments distinctly manipulate *E_MSH_* of *Mtb*, varied redox oscillatory behavior could simply be a consequence of differences in co-localization kinetics of *Mtb* strains within sub-vacuolar compartments over time. Alternatively, phenotypic variations such as uptake rate and intramacrophage growth kinetics may influence the *E_MSH_* of these strains. While the molecular mechanisms behind these redox variations and their influence on evolution of drug-resistance and fitness await further investigation, several studies have documented strain-specific differences in bacterial and host gene expression during infection [55], [56], and resistance to redox stress [57], [58].

Numerous studies using oxidant-sensitive dyes have demonstrated that bactericidal antibiotics, regardless of their target, exert toxicity by stimulating Fenton-catalyzed ROS production [59]–[61]. However, two recent studies demonstrate that measurements using dyes may be inconclusive owing to their non-specificity and that the bactericidal potential of antibiotics does not correlate with ROS generation *in vitro* [62], [63]. Furthermore, to the best of our knowledge, the contribution of antibiotics-stimulated oxidative stress in the physiological context of infection has not been evaluated. In this context, Mrx1-roGFP2 allowed us to investigate the redox-basis of drug action in *Mtb*. We show that *Mtb* maintains *E_MSH_* in response to antibiotic exposure (except in case of CFZ) during *in vitro* growth, whereas a significant oxidative shift was induced by all drugs during intramacrophage growth. This data along with the antibiotic sensitivity displayed by the MSH-deficient strains [21] suggest that MSH biosynthesis or recycling efficiently buffers toxicity associated with drugs during normal growing conditions. In this regard, mycothiol-S-conjugate detoxification system (Mca) maintains cytoplasmic MSH levels upon antibiotic exposure by rapidly converting MSH-antibiotic adducts to mercapturic acids and excreting them into the culture media [44]. Another *Mtb* antioxidant, ERG, was found to be over-expressed in MSH-mutants, however, it does not provide protection against antibiotics [64].

Importantly, we show that the antibiotic-mediated increase in *E_MSH_*-oxidized precedes bacterial death inside infected macrophages. Oxidative shift in *E_MSH_* was substantial at time points earlier than those at which drug antimycobacterial activities were achieved. Moreover, INH-resistant clinical strains remained uninfluenced by INH-mediated oxidative changes in *E_MSH_* during infection, thus supporting our conclusions. Our results suggest that an active cooperation between host factors and antibiotics could disrupt intramycobacterial *E_MSH_* during chemotherapy. This is in agreement with a recent study demonstrating the role of anti-TB drugs in inducing host ROS production and potentiating mycobactericidal activity by delivering *Mtb* into lysosomal and autophagosomal compartments [65]. Since lysosomes and autophagosomes predominantly contain *Mtb* cells with an oxidized *E_MSH_*, our findings present a novel insight into the mechanism of antibiotic action during infection. Together, our data suggest that direct effects of antibiotics on *Mtb* physiology and on host innate immune mechanisms such as autophagy and phagosomal maturation, jointly eliminate *Mtb* bacilli by stimulating overwhelming intramycobacterial oxidative stress *in vivo*. In line with this hypothesis, we show that treatment with autophagy inducer (rapamycin) significantly augments mycobactericidal activities of antibiotics during infection. While MSH has been shown to be dispensable for growth of *Mtb* in mice [66], future experiments should target MSH levels during antibiotic treatment to understand its function in modulating bacterial killing during antimicrobial therapy *in vivo*, where the heterogeneity in *E_MSH_* may be one of the critical determinants of persistence and drug tolerance. These exciting hypotheses can now be investigated by integrating Mrx1-roGFP2 technology with high-resolution live cell profiling of intramycobacterial *E_MSH_* at the single-cell level during infection.

How do these findings relate to human TB? Within human host, *Mtb* persists in a state of drug unresponsiveness in oxygen-depleted and lipid-rich granulomas [67]. The center of TB granuloma is hypoxic (3 mM Hg) and contains lipid-laden foamy macrophages and free fatty acids released from necrotic macrophages [67], [68]. Recent studies suggest that β-oxidation of host fatty acids by *Mtb* as the primary carbon source in an O_2_-deficient environment leads to massive accumulation of NADH/NADPH, which generates intrabacterial reductive stress in persisting cells during infection [10], [69]. Work presented here demonstrates that drug tolerance observed during persistence may be mediated by increased reductive capacity. Further strengthening this connection are recent findings demonstrating elimination of *Mtb* persisters by drugs which become active under reductive stress (*e.g.*, metranidazole) or generate overwhelming ROS (*e.g.*, CFZ) [70], [71]. Another common clinical observation is that *Mtb* genetically resistant to only a subset of anti-TB drugs resists clearance from all other antibiotics and thus, survive combination drug therapy [72]. One interesting possibility revealed by this work is that redox heterogeneity within genetically drug-resistant clinical isolates (MDR/XDR) provides a subpopulation that tolerates antibiotics against which bacteria are genetically susceptible. These understandings shed new light on drug resistant mechanisms and suggest that novel approaches that disrupt redox metabolism in *Mtb* may significantly impact eradication of both genetic and phenotypic drug resistant populations.

In summary, we have developed a genetically encoded, fluorescent reporter capable of monitoring intrabacterial *E_MSH_* within macrophages. We anticipate that Mrx1-roGFP2 will play an important role in high content screening of small-molecule inhibitors of intrabacterial redox homeostasis. Based on this work, one can readily imagine development of new biosensors to measure the redox state of specific and unusual redox thiols, such as BSH, trypanothione, ovothiol A found in a variety of pathogenic organisms. Finally, macrophage-induced redox heterogeneity and its connection to drug sensitivity may be relevant to other intracellular pathogens. For example, the macrophage environment induces expression of genes responsible for antioxidant production and drug-tolerance in *Legionella pneumophilla* [73]. Thus, our findings may have relevance to several intracellular pathogens causing chronic and relapsing infections where persistence and tolerance pose challenges for treatment.

## Materials and Methods

### Cloning, purification, and biochemical characterization of the Mrx1-roGFP2

The roGFP2 containing vector was obtained from Tobias P. Dick [24]. The roGFP2 open reading frame (ORF) was released using *Nco*I and *Hind*III and was cloned downstream of *hsp60* promoter into similarly digested *E. coli*-mycobacterial shuttle vector, pMV762 [74] to generate pMV762-roGFP2. Mrx1-roGFP2 biosensor construct was generated by fusing the coding sequence of *Mtb* Mrx1 (Rv3198A) with roGFP2 having a 30-amino acid linker (GGSGG)_6_ between the two genes. Mrx1 coding sequence was amplified from *Mtb* genome (Forward primer: 5′ ATGCCCATGGTGATCACCGCTGCG 3′. Reverse primer: 5′ ATGCACTAGTACCCGCGATCTTTAC 3′). Cysteine mutations in Mrx1 coding sequence were introduced by site-directed mutagenesis as described [11]. Primers used were: Mrx1 (AGYC) forward primer: 5′ CTATACGACATCATGG**GCT**GGCTATTGCCTTCGAC 3′, reverse primer: 5′ GTCGAAGGCAATAGCC**AGC**CCATGATGTCGTATAG 3′, Mrx1 (CGYA) forward primer: 5′ CATCATGGTGTGGCTAT**GCC**CTTCGACTCAAAACAG 3′, reverse primer: 5′ CTGTTTTGAGTCGAAG**GGC**ATAGCCACACCATGATG 3′. All the fusion constructs were sub cloned into the expression vector pET28b (Novagen), expressed in the *E. coli* strain BL21 DE3 (Stratagene), and fusion proteins were purified via hexahistidine affinity chromatography as described [11]. Aerobically purified Mrx1-roGFP2 and ro-GFP2 were found to be in the fully oxidized state. To study the effect of various oxidants *in vitro*, the roGFP2 fusion proteins (1 µM) were first reduced with 10 mM DTT for 30 min on ice and desalted with Zeba Desalt spin columns (Pierce Biotechnology). *In vitro* measurements using various roGFP2 variants were performed on SpectraMax M3 microplate reader.

### Mtr electron transfer assay

*Mtb mtr* ORF (Rv3198A) was cloned into pET28b (Novagen) and expressed in the *E. coli* strain BL21 DE3. Purification of Mtr protein was performed as described in the earlier section. Mycothiol is purchased from JEMA Biosciences, San Diego, CA, USA. To perform Mtr electron transfer assay, a mixture of 2.5 µM purified Mtr, 250 µM MSSM and 500 µM NADPH was prepared in 50 mM HEPES pH 8.0 in a 96-well plate. In the control reaction, Mtr was absent. The mixture was incubated at 37°C and consumption of NADPH was monitored at 340 nm for 60 min. To check the specificity of roGFP2 fusion proteins towards MSH, Mtr assay mixture was prepared as described above. After incubation at 37°C for 30 min, oxidized roGFP2 fusion proteins (1 µM) were added to the mix. Ratiometric sensor response was monitored for 200 min. A control reaction without MSSM was included.

### Sensitivity of Mrx1-roGFP2 towards changes in OxD_MSH_

Pre-reduced uncoupled roGFP2 and Mrx1-roGFP2 (1 µM) were incubated with mycothiol solutions (1 mM total) containing increasing fractions of MSSM. The total concentration of MSH (MSH _total_) refers to MSH equivalents i. e MSH_total_ = [MSH]+2[MSSM]. OxD_MSH_ is the fraction of MSH _total_ that exists as [MSSM] and can be conveniently calculated using the following formula:
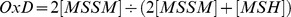
Reduced from of mycothiol (MSH) was obtained by reducing MSSM with immobilized TCEP disulfide reducing gel (Thermo Scientific) under anaerobic conditions as per manufacturer's instructions.

### Miscellaneous procedures

Detailed Materials and Methods are provided in the supporting information.

## Supporting Information

Figure S1 (A) 1 µM of aerobically purified Mrx1-roGFP2 (oxidized) was reduced by 10 mM DTT and fluorescence intensity was measured using spectrofluorometer. Note an increase in the fluorescence intensity ∼390 nm and a concomitant decrease at ∼490 nm in the oxidized Mrx1-roGFP2, whereas reverse is observed upon reduction with DTT. (B) Recombinant Mrx1-roGFP2 was diluted into phosphate buffer with different pH values. For protein reduction, 10 mM DTT was added. After 20 min of incubation at room temperature, the excitation ratio at 390 nm and 490 nm was calculated. The ratio of fully oxidized and fully reduced Mrx1-roGFP2 is depicted at different pH values. (C) 1 µM of Mrx1-roGFP2 was treated with DTT_red_:DTT_oxd_ solutions (final concentration of DTT_red_+DTT_oxd_ ≥10 mM in PBS) that had the redox potentials ranging from −400 to −150 mV. The resulting change in the Mrx1-roGFP2 ratios were plotted against the equivalent redox potential values and data was fit to a titration curve. As seen in the curve, midpoint potential of roGFP2 (−280 mV) is maintained in Mrx1-roGFP2. (D) Mycothiol reductase (Mtr) assay using MSSM as a substrate. NADPH dependent reduction of MSSM by Mtr was monitored by tracking the rate of NADPH oxidation to NADP^+^. The consumption of NADPH at 340 nm is shown.
(TIF)

Figure S2 (A) Oxidized Mrx1-roGFP2 protein was treated with 5 mM of ERG and 10 mM DTT under anaerobic conditions and ratiometric sensor response was measured at indicated time points. (B) 1 µM of pre reduced Mrx1-roGFP2 was exposed to different concentrations of H_2_O_2_ and the ratio change after 60 sec was measured. (C) Exponentially growing *Msm* cells expressing Mrx1-roGFP2 were treated with 250 µM of oxidants (as shown in the figure) and ratiometric response was measured. (D) Standard curve for the determination of intracellular levels of Mrx1-roGFP2. Indicated concentrations of purified Mrx1-roGFP2 protein were subjected to immunoblot analysis using antibodies against GFP. 10 ml of *Msm* cells over-expressing Mrx1-roGFP2 were grown till an OD _600_ nm of 0.8, harvested, and 15 µg of cell free extract was analyzed for the expression of Mrx1-roGFP2 by immunoblot analysis using antibodies against GFP. The band intensities were quantified by ImageJ software. Based on the standard curve, we calculated the amount of Mrx1-roGFP2 expressed inside a single *Msm* cell. (E) Mrx1 overexpression has no effect on the sensitivity of *Msm* towards H_2_O_2_. *Msm*, *MsmΔmshA* and *MsmΔmtr* strains with and without Mrx1-roGFP2 overexpression were exposed to H_2_O_2_ for 2 h and plated for CFU. Different concentrations of H_2_O_2_ (20 mM for wt *Msm*, 5 mM for *MsmΔmshA*, and 10 mM for *MsmΔmtr*) were chosen due to differences in the sensitivity of each strain towards peroxide stress. Error bars indicate standard deviations from the mean. Data shown is the average of three independent experiments performed in triplicate.
(TIF)

Figure S3 (A) Alamar blue microplate assay with H37Rv and H37Rv expressing Mrx1-roGFP2. 5×10^5^ bacilli per ml were taken and after incubation for 5 days 1× alamar blue was added. After further incubation for 1 day, fluorescence readings were taken (Ex 530 nm, Em 590 nm). (B) Cultures of H37Rv and H37Rv expressing Mrx1-roGFP2 were synchronized to OD_600 nm_ = 0.1–0.15 and grown in 7H9-OADC media. Culture density (OD_600 nm_) was measured at the indicated time points. (C) *Mtb* H37Rv was given different treatments (10 mM NEM was added prior to or after 1 mM H_2_O_2_/4% PFA) and the resulting redox state of the Mrx1-roGFP2 was determined by flow cytometry. Note the increase in Mrx1-roGFP2 ratios upon exposure to PFA and H_2_O_2_ prior to NEM treatment. NEM treatment effectively clamps intracellular redox state of Mrx1-roGFP2 thiols, thereby preventing oxidation artifacts induced during fixation of *Mtb* cells by PFA. * p-values<0.01. Results are representative of two independent experiments showing similar results.
(TIF)

Figure S4 A) *Mtb* H37Rv Mrx1-roGFP2 was treated with 1 mM CHP or 40 mM DTT and ratiometric sensor response was measured. (B) *Mtb* H37Rv expressing Mrx1-roGFP2 was treated with varying concentrations of H_2_O_2_ for 10 min and ratiometric sensor response was measured. (C) *Mtb* H37Rv Mrx1-roGFP2 was treated with the 5 mM H_2_O_2_ and the ratiometric sensor response was measured. Error bars represent standard deviations from the mean. Data are representative of at least three independent experiments.
(TIF)

Figure S5 (A) *Mtb* H37Rv expressing Mrx1-roGFP2 was treated with 10 mM DTT (for 100% Mrx1-roGFP2 reduction), 1 mM cumene hydroperoxide (CHP) (for 100% Mrx1-roGP2 oxidation) and DTT_red_:DTT_oxd_ solutions (final concentration of DTT_red_+DTT_oxd_≥10 mM in PBS) that had the redox potentials ranging from −330 to −195 mV (see SI *Materials and Methods*). The resulting change in the Mrx1-roGFP2 ratios were normalized to the ratio with 10 mM DTT_red_ giving 0% oxidation and ratio with 1 mM CHP giving 100% oxidation. Apparent redox potential values of Mrx1-roGFP2 were determined by plotting average Mrx1-roGFP2 ratios versus the equivalent redox potential values and fitting the data to a titration curve. (B and C) MOI dependent changes in intrabacterial *E_MSH_* during infection. THP-1 cells were infected with H37Rv expressing Mrx1-roGFP2 at a moi of (B) 1 and (C) 10. At indicated time points, cells were treated with NEM-PFA and 30,000 infected macrophages were analyzed by flow cytometry and intramycobacterial *E_MSH_* was measured as described in SI *Materials and Methods*. The percentage of bacilli in each subpopulation was calculated and plotted as a bar graph. Error bars represent standard deviations from the mean. Data is representative of three independent experiments.
(TIF)

Figure S6 (A) iNOS inhibition influences intramycobacterial *E_MSH_* in immune-activated RAW 264.7 macrophages. IFN-γ/LPS activated RAW 264.7 macrophages were infected with H37Rv expressing Mrx1-roGFP2 (moi: 10) and subsequently treated with 1 mM NMLA. At 48 h p.i., cells were treated with NEM-PFA and 30,000 infected macrophages were analyzed by flow cytometry and intramycobacterial *E_MSH_* was measured as described earlier. The percentage of bacilli in each subpopulation was calculated and plotted as a bar graph. * p<0.01. Error bars represent standard deviations from the mean. Data is representative of three independent experiments. (B) *E_MSH_* of *Mtb* H37Rv grown *in vitro*. *Mtb* H37Rv was grown in 7H9 medium till exponential phase. Cells were treated with NEM and fixed with PFA followed by analysis at the single cell level by confocal microscopy. Ratiometric imaging was performed using 405 and 488 nm lasers. False color ratio image of mid log phase *Mtb* H37Rv expressing Mrx1-roGFP2 was generated by using the lookup table “Fire” of ImageJ (see SI *Materials and Methods*). Also shown is the color bar displaying a range of 405/488 nm ratios from 0 to 1. Analysis of ∼100 bacilli revealed that majority of bacteria (∼80%) has similar Mrx1-roGFP2 ratios, suggesting that *in vitro* grown *Mtb* are predominantly redox homogeneous. Data is representative of three independent experiments performed in triplicate.
(TIF)

Figure S7 Intramycobacterial heterogeneity in *E_MSH_* within early endosomes and lysosomes. THP-1 cells were infected with H37Rv expressing Mrx1-roGFP2 (moi: 10). At each time point infected cells were treated by NEM-PFA. Cells were then stained for Rab5 and Cathepsin D and analyzed by confocal microscopy for measuring ratiometric sensor response in *Mtb* co-localized within sub-vacuolar compartments. Co-localization of *Mtb* H37Rv in (A) endosomes and (B) lysosomes. In the merge panel, green and red indicates the bacilli and the compartment markers, respectively. The overlap is demonstrated in the merge images, where yellow indicates a positive correlation. False color ratio images were generated as described in SI *Materials and Methods*. Small dashed line boxes indicate co-localized bacilli and large solid line boxes represent the enlarged view of the one of the co-localized bacilli. Numbers represent *E_MSH_* in millivolts. *E_MSH_* of co-localized bacilli (≥50) is calculated and distribution is shown in scatter plot. Each point on the plot represents a bacterium. Bar represents mean values. p-values were calculated by one way ANOVA followed by Tukey's HSD statistical test (* p<0.01). Percentage of bacilli in each subpopulation is represented as a stacked bar graph. Color bar corresponds to the 405/488 nm ratios ranging from 0 to 1. Data shown is the representative of at least three independent experiments.
(TIF)

Figure S8 THP-1 cells were infected with H37Rv expressing Mrx1-roGFP2. 24 h p.i., cells were treated with NEM and PFA, followed by flow cytometry. In a parallel experiment, infected macrophages were lysed and redox heterogeneity within released *Mtb* cells was analyzed by flow cytometry. This result clearly demonstrates that NEM-treated *Mtb* maintains macrophage induced heterogeneity in *E_MSH_* after its liberation from macrophages. Although we have effectively blocked Mrx1-roGFP2 redox state by NEM, previous studies showed that host-induced antibiotic tolerance was preserved in *Mtb* even after its release from macrophages and subsequent culturing in 7H9 medium *in vitro* [49]. Similarly, we and others have previously reported that *Mtb* maintains host-induced changes in metabolism and redox state in 7H9 medium for a few generations [10], [69]. This allowed us to stain macrophage conditioned *Mtb* with Pi for determining membrane integrity status post-antibiotic treatment.
(TIF)

Table S1 Drug-resistance patterns, clade identity, and *E_MSH_* for H37Rv and different field isolates used in this study.
(TIF)

Text S1 Supporting text. This file contains detailed methods, including *in vitro* redox calibration curve using Mrx1-roGFP2 protein, culture conditions for mammalian and bacterial cells, macrophage infection, quantification of Mrx1-roGFP2 expression, *in vitro* redox calibration curve using *Mtb* H37Rv, measurement of *E_MSH_* by confocal and flow cytometry, Alamar blue and growth assays, Pi staining, effect of rapamycin and DTT on anti-TB drugs activity, and statistical analysis.
(DOCX)
