# Supplementary figures and images for: Reengineering Redox Sensitive GFP to Measure Mycothiol Redox Potential of Mycobacterium tuberculosis during Infection
Source: PLoS Pathog. 2014 Jan 30;10(1):e1003902. doi: 10.1371/journal.ppat.1003902 (PMC3907381; doi:10.1371/journal.ppat.1003902)

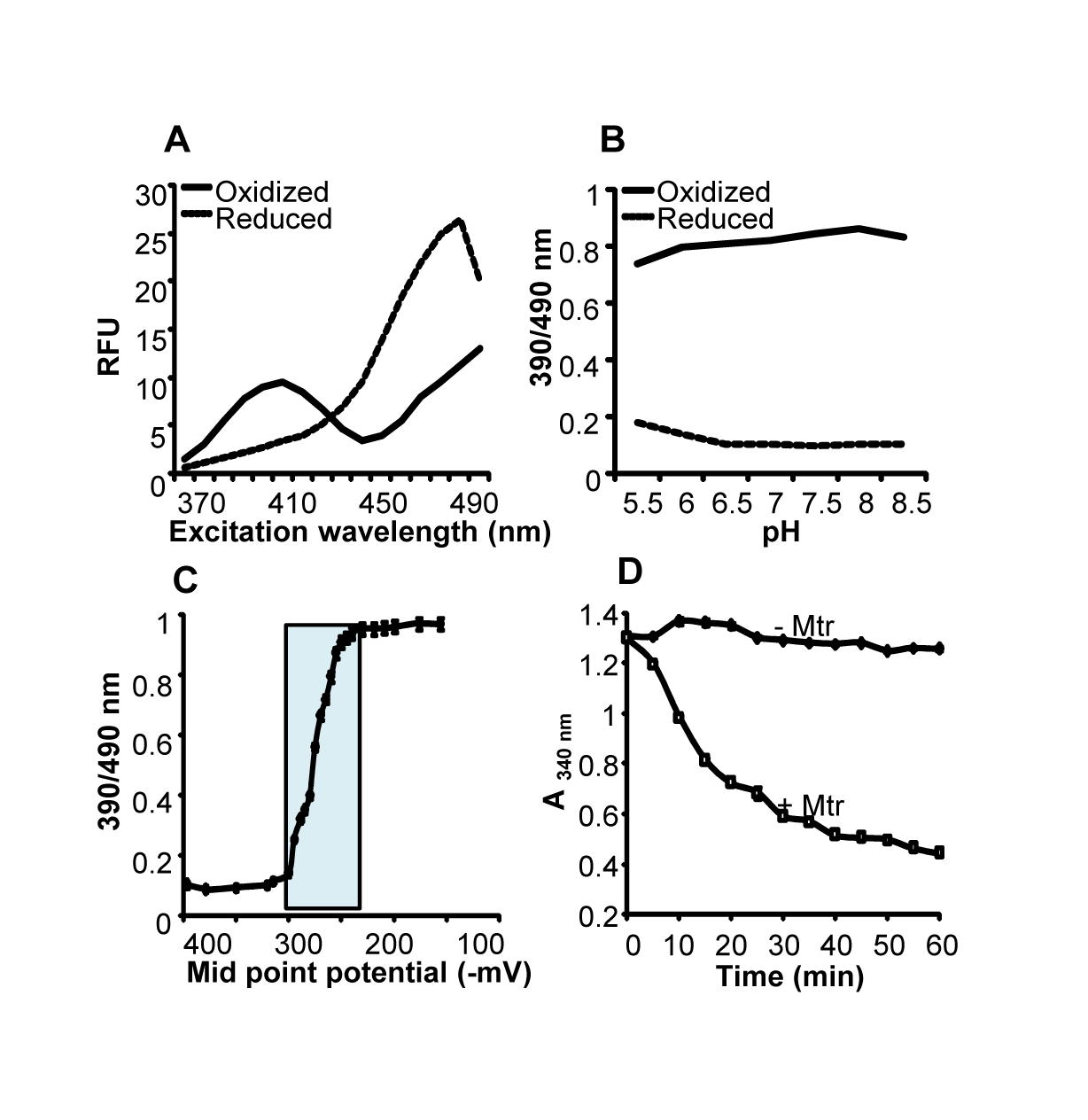

Supplement: Figure S1 — (A) 1 µM of aerobically purified Mrx1-roGFP2 (oxidized) was reduced by 10 mM DTT and fluorescence intensity was measured using spectrofluorometer. Note an increase in the fluorescence intensity ∼390 nm and a concomitant decrease at ∼490 nm in the oxidized Mrx1-roGFP2, whereas reverse is observed upon reduction with DTT. (B) Recombinant Mrx1-roGFP2 was diluted into phosphate buffer with different pH values. For protein reduction, 10 mM DTT was added. After 20 min of incubation at room temperature, the excitation ratio at 390 nm and 490 nm was calculated. The ratio of fully oxidized and fully reduced Mrx1-roGFP2 is depicted at different pH values. (C) 1 µM of Mrx1-roGFP2 was treated with DTTred:DTToxd solutions (final concentration of DTTred+DTToxd ≥10 mM in PBS) that had the redox potentials ranging from −400 to −150 mV. The resulting change in the Mrx1-roGFP2 ratios were plotted against the equivalent redox potential values and data was fit to a titration curve. As seen in the curve, midpoint potential of roGFP2 (−280 mV) is maintained in Mrx1-roGFP2. (D) Mycothiol reductase (Mtr) assay using MSSM as a substrate. NADPH dependent reduction of MSSM by Mtr was monitored by tracking the rate of NADPH oxidation to NADP+. The consumption of NADPH at 340 nm is shown. (TIF) [file ppat.1003902.s001.tif]

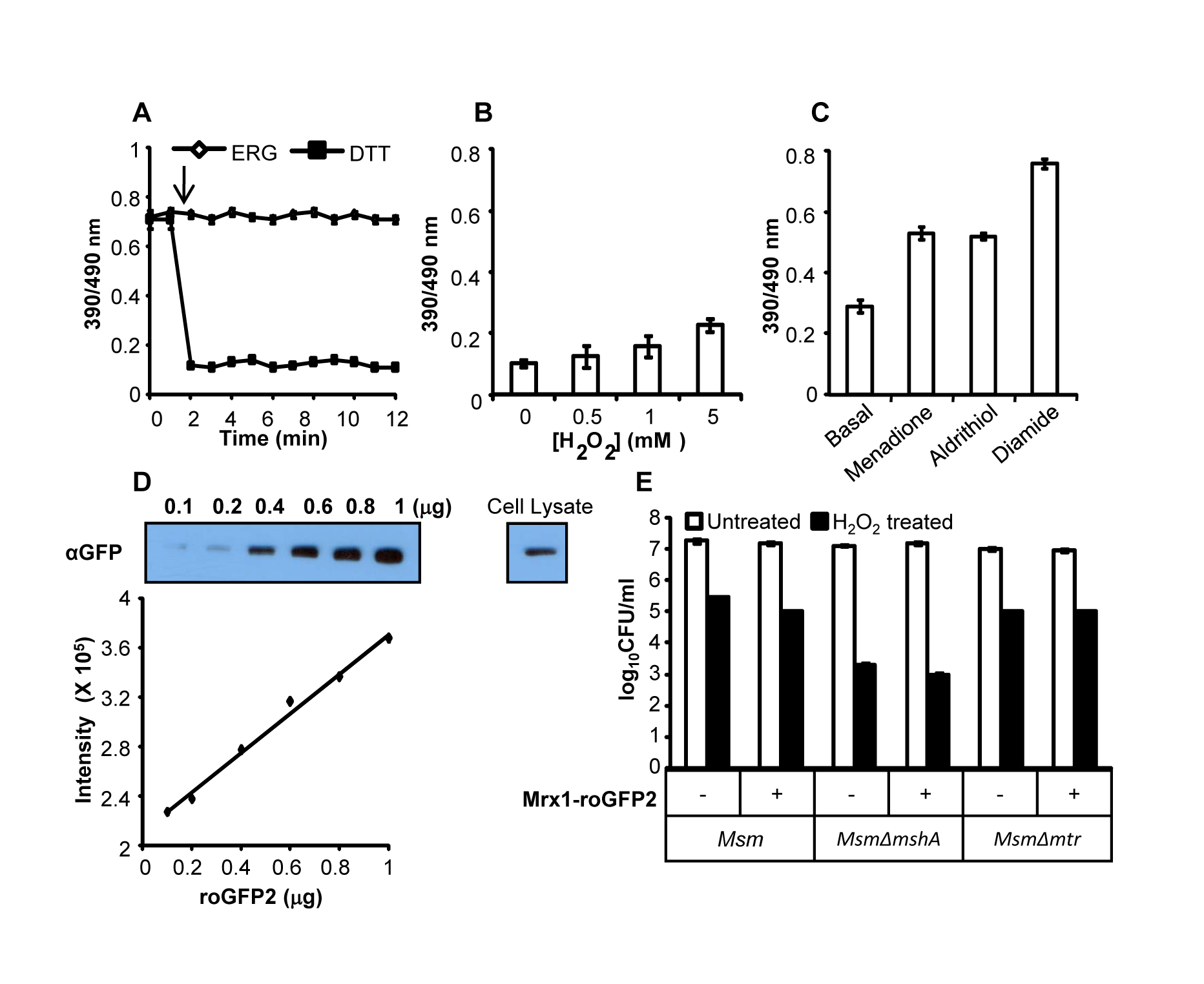

Supplement: Figure S2 — (A) Oxidized Mrx1-roGFP2 protein was treated with 5 mM of ERG and 10 mM DTT under anaerobic conditions and ratiometric sensor response was measured at indicated time points. (B) 1 µM of pre reduced Mrx1-roGFP2 was exposed to different concentrations of H2O2 and the ratio change after 60 sec was measured. (C) Exponentially growing Msm cells expressing Mrx1-roGFP2 were treated with 250 µM of oxidants (as shown in the figure) and ratiometric response was measured. (D) Standard curve for the determination of intracellular levels of Mrx1-roGFP2. Indicated concentrations of purified Mrx1-roGFP2 protein were subjected to immunoblot analysis using antibodies against GFP. 10 ml of Msm cells over-expressing Mrx1-roGFP2 were grown till an OD 600 nm of 0.8, harvested, and 15 µg of cell free extract was analyzed for the expression of Mrx1-roGFP2 by immunoblot analysis using antibodies against GFP. The band intensities were quantified by ImageJ software. Based on the standard curve, we calculated the amount of Mrx1-roGFP2 expressed inside a single Msm cell. (E) Mrx1 overexpression has no effect on the sensitivity of Msm towards H2O2. Msm, MsmΔmshA and MsmΔmtr strains with and without Mrx1-roGFP2 overexpression were exposed to H2O2 for 2 h and plated for CFU. Different concentrations of H2O2 (20 mM for wt Msm, 5 mM for MsmΔmshA, and 10 mM for MsmΔmtr) were chosen due to differences in the sensitivity of each strain towards peroxide stress. Error bars indicate standard deviations from the mean. Data shown is the average of three independent experiments performed in triplicate. (TIF) [file ppat.1003902.s002.tif]

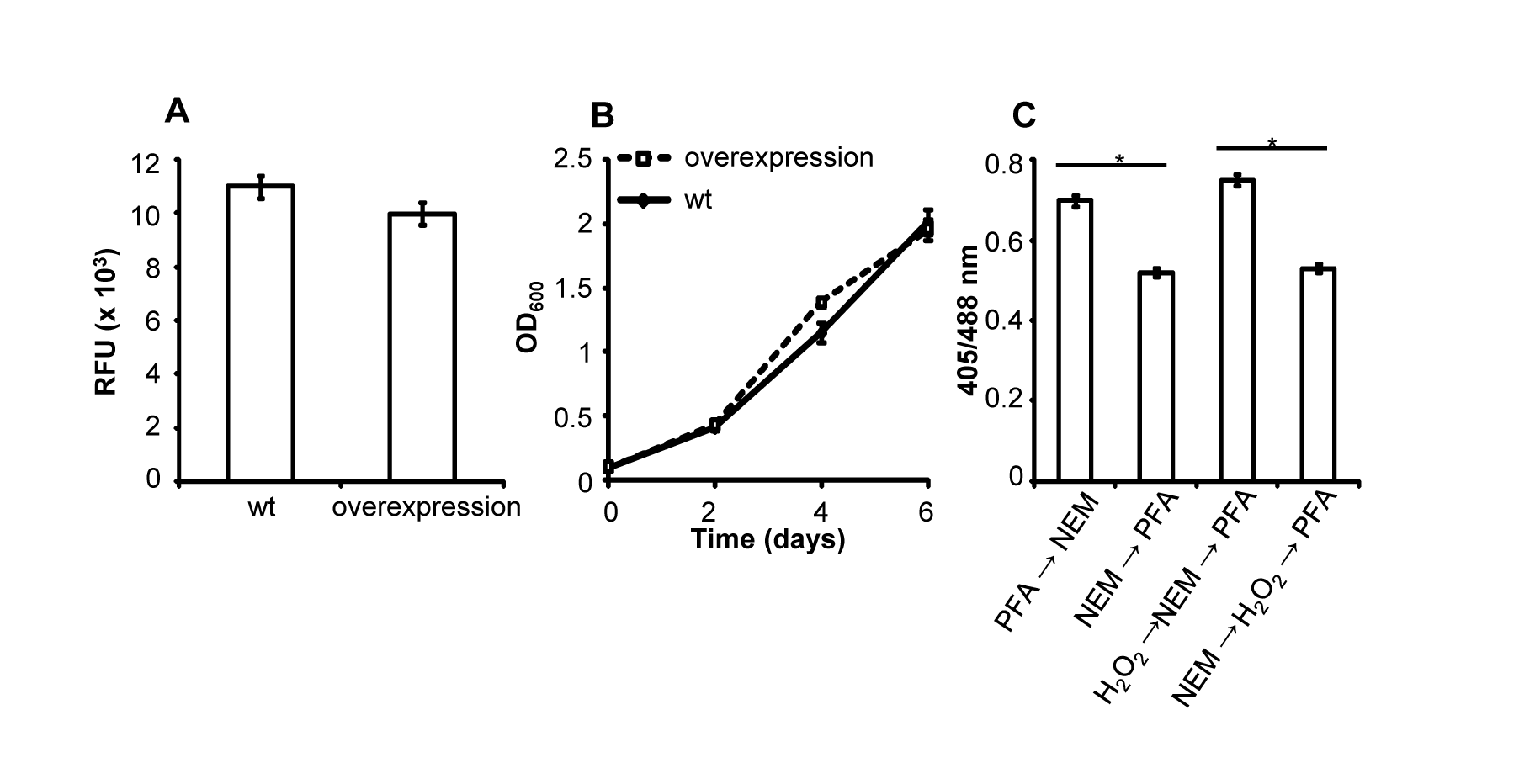

Supplement: Figure S3 — (A) Alamar blue microplate assay with H37Rv and H37Rv expressing Mrx1-roGFP2. 5×10^5^ bacilli per ml were taken and after incubation for 5 days 1× alamar blue was added. After further incubation for 1 day, fluorescence readings were taken (Ex 530 nm, Em 590 nm). (B) Cultures of H37Rv and H37Rv expressing Mrx1-roGFP2 were synchronized to OD600 nm = 0.1–0.15 and grown in 7H9-OADC media. Culture density (OD600 nm) was measured at the indicated time points. (C) Mtb H37Rv was given different treatments (10 mM NEM was added prior to or after 1 mM H2O2/4% PFA) and the resulting redox state of the Mrx1-roGFP2 was determined by flow cytometry. Note the increase in Mrx1-roGFP2 ratios upon exposure to PFA and H2O2 prior to NEM treatment. NEM treatment effectively clamps intracellular redox state of Mrx1-roGFP2 thiols, thereby preventing oxidation artifacts induced during fixation of Mtb cells by PFA. * p-values<0.01. Results are representative of two independent experiments showing similar results. (TIF) [file ppat.1003902.s003.tif]

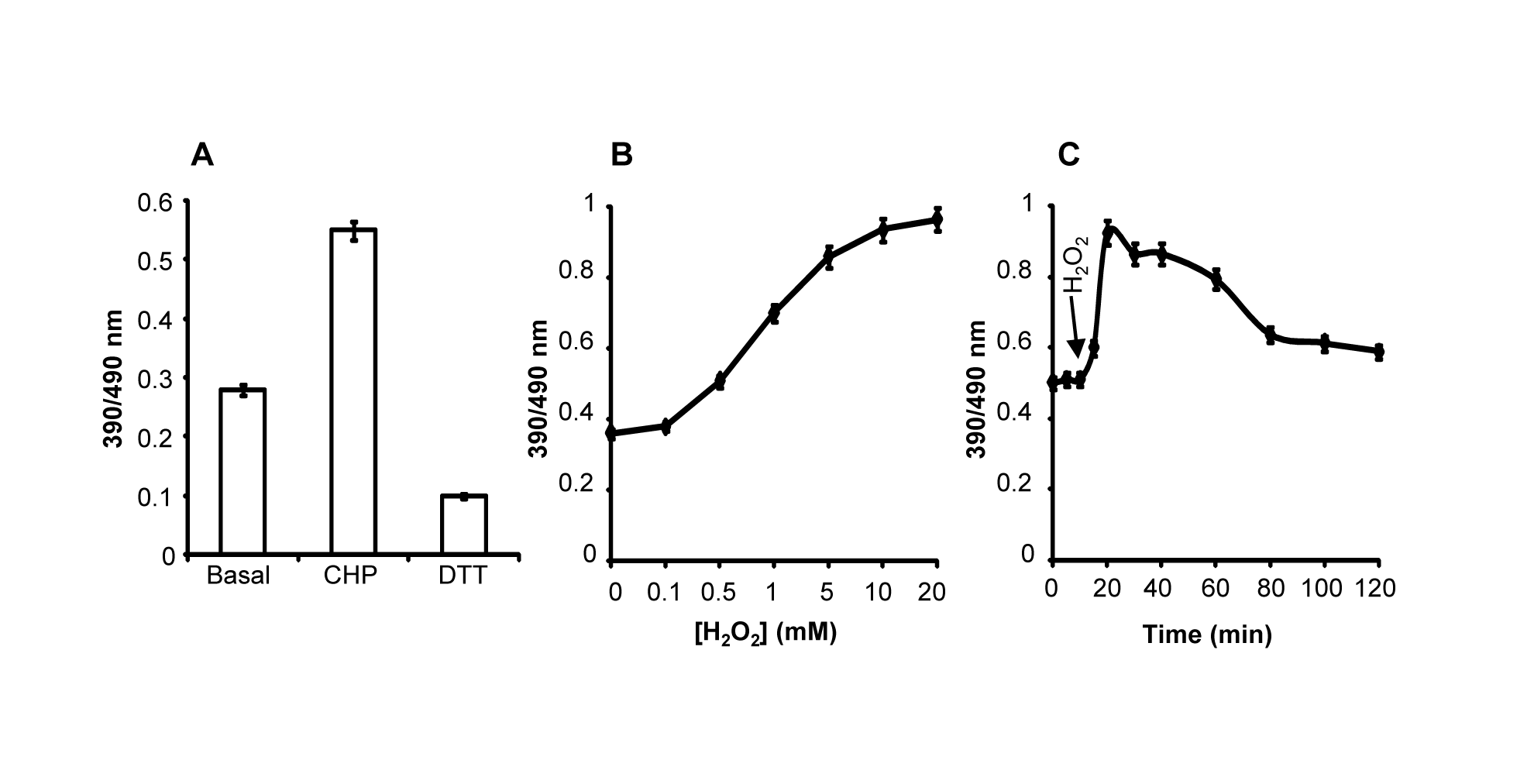

Supplement: Figure S4 — A) Mtb H37Rv Mrx1-roGFP2 was treated with 1 mM CHP or 40 mM DTT and ratiometric sensor response was measured. (B) Mtb H37Rv expressing Mrx1-roGFP2 was treated with varying concentrations of H2O2 for 10 min and ratiometric sensor response was measured. (C) Mtb H37Rv Mrx1-roGFP2 was treated with the 5 mM H2O2 and the ratiometric sensor response was measured. Error bars represent standard deviations from the mean. Data are representative of at least three independent experiments. (TIF) [file ppat.1003902.s004.tif]

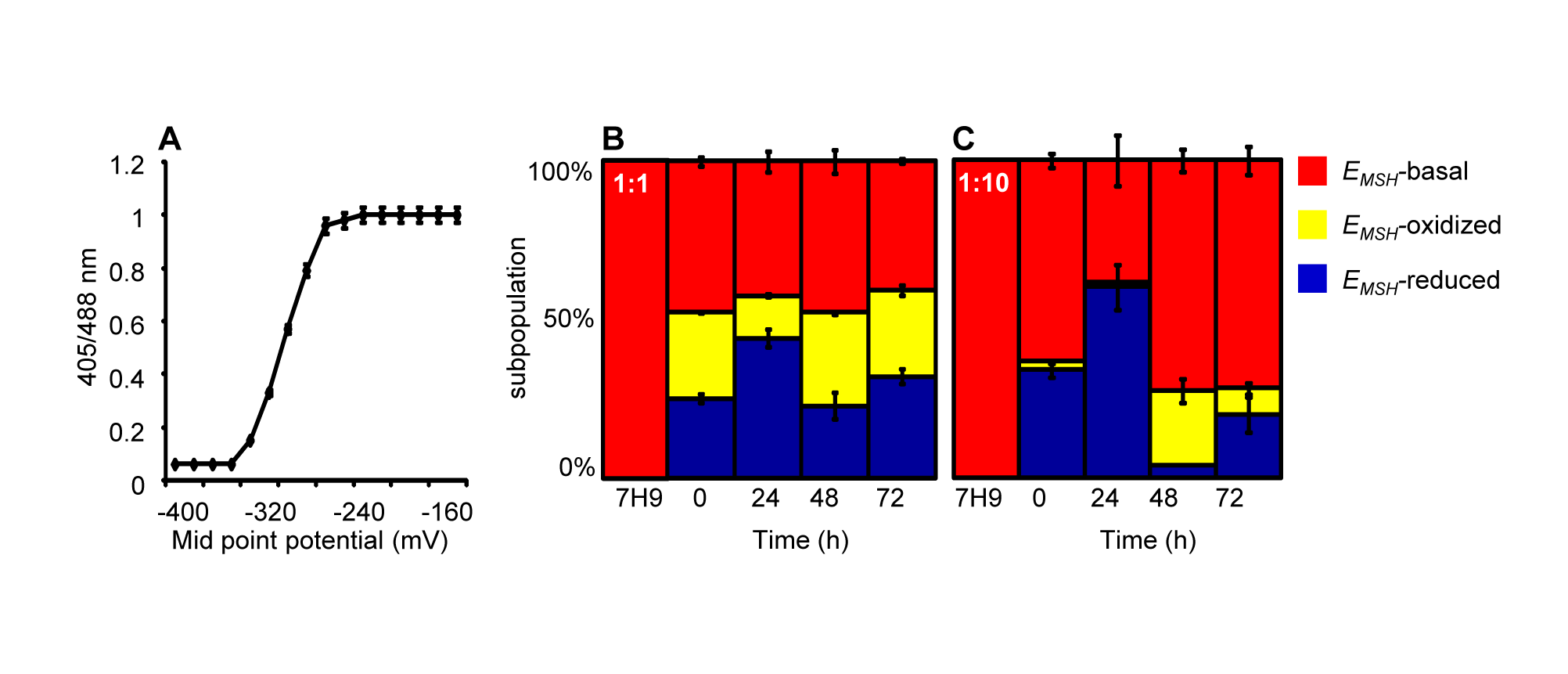

Supplement: Figure S5 — (A) Mtb H37Rv expressing Mrx1-roGFP2 was treated with 10 mM DTT (for 100% Mrx1-roGFP2 reduction), 1 mM cumene hydroperoxide (CHP) (for 100% Mrx1-roGP2 oxidation) and DTTred:DTToxd solutions (final concentration of DTTred+DTToxd≥10 mM in PBS) that had the redox potentials ranging from −330 to −195 mV (see SI Materials and Methods ). The resulting change in the Mrx1-roGFP2 ratios were normalized to the ratio with 10 mM DTTred giving 0% oxidation and ratio with 1 mM CHP giving 100% oxidation. Apparent redox potential values of Mrx1-roGFP2 were determined by plotting average Mrx1-roGFP2 ratios versus the equivalent redox potential values and fitting the data to a titration curve. (B and C) MOI dependent changes in intrabacterial EMSH during infection. THP-1 cells were infected with H37Rv expressing Mrx1-roGFP2 at a moi of (B) 1 and (C) 10. At indicated time points, cells were treated with NEM-PFA and 30,000 infected macrophages were analyzed by flow cytometry and intramycobacterial EMSH was measured as described in SI Materials and Methods . The percentage of bacilli in each subpopulation was calculated and plotted as a bar graph. Error bars represent standard deviations from the mean. Data is representative of three independent experiments. (TIF) [file ppat.1003902.s005.tif]

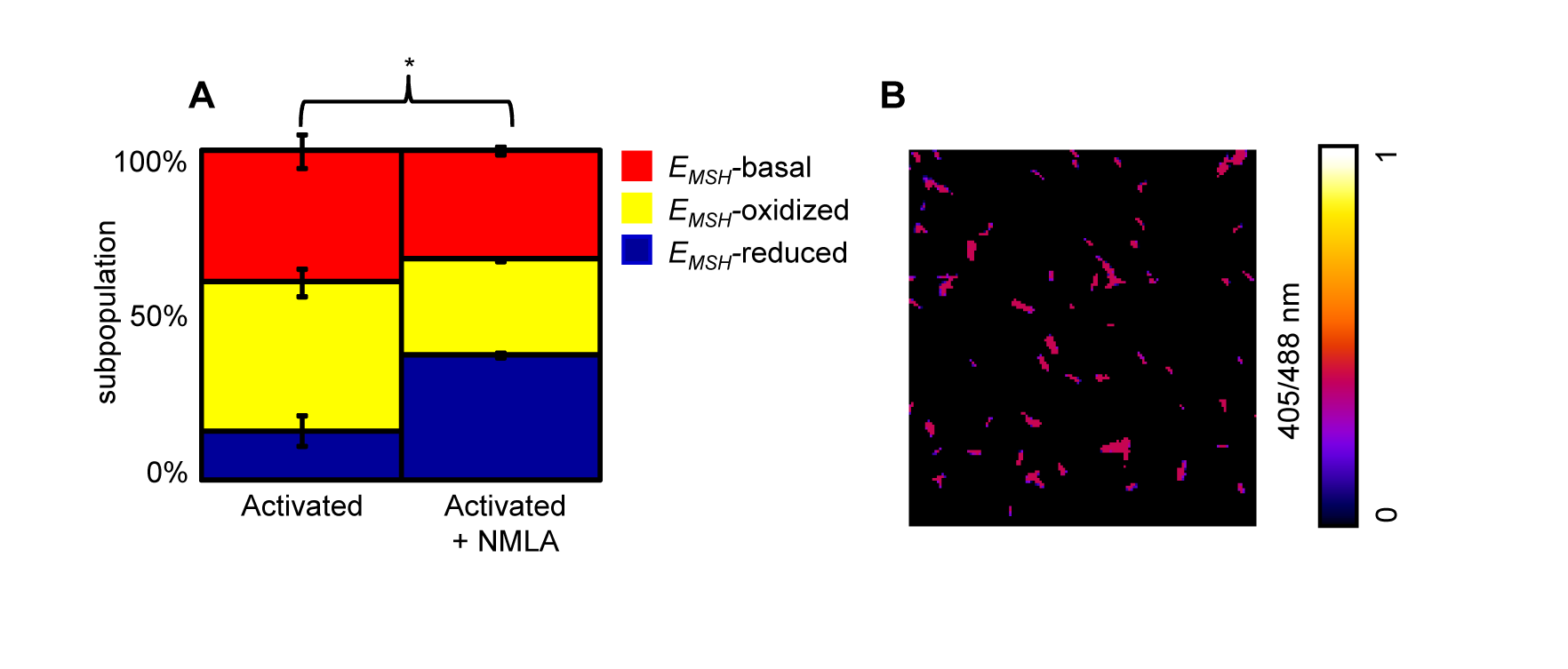

Supplement: Figure S6 — (A) iNOS inhibition influences intramycobacterial EMSH in immune-activated RAW 264.7 macrophages. IFN-γ/LPS activated RAW 264.7 macrophages were infected with H37Rv expressing Mrx1-roGFP2 (moi: 10) and subsequently treated with 1 mM NMLA. At 48 h p.i., cells were treated with NEM-PFA and 30,000 infected macrophages were analyzed by flow cytometry and intramycobacterial EMSH was measured as described earlier. The percentage of bacilli in each subpopulation was calculated and plotted as a bar graph. * p<0.01. Error bars represent standard deviations from the mean. Data is representative of three independent experiments. (B) EMSH of Mtb H37Rv grown in vitro. Mtb H37Rv was grown in 7H9 medium till exponential phase. Cells were treated with NEM and fixed with PFA followed by analysis at the single cell level by confocal microscopy. Ratiometric imaging was performed using 405 and 488 nm lasers. False color ratio image of mid log phase Mtb H37Rv expressing Mrx1-roGFP2 was generated by using the lookup table “Fire” of ImageJ (see SI Materials and Methods ). Also shown is the color bar displaying a range of 405/488 nm ratios from 0 to 1. Analysis of ∼100 bacilli revealed that majority of bacteria (∼80%) has similar Mrx1-roGFP2 ratios, suggesting that in vitro grown Mtb are predominantly redox homogeneous. Data is representative of three independent experiments performed in triplicate. (TIF) [file ppat.1003902.s006.tif]

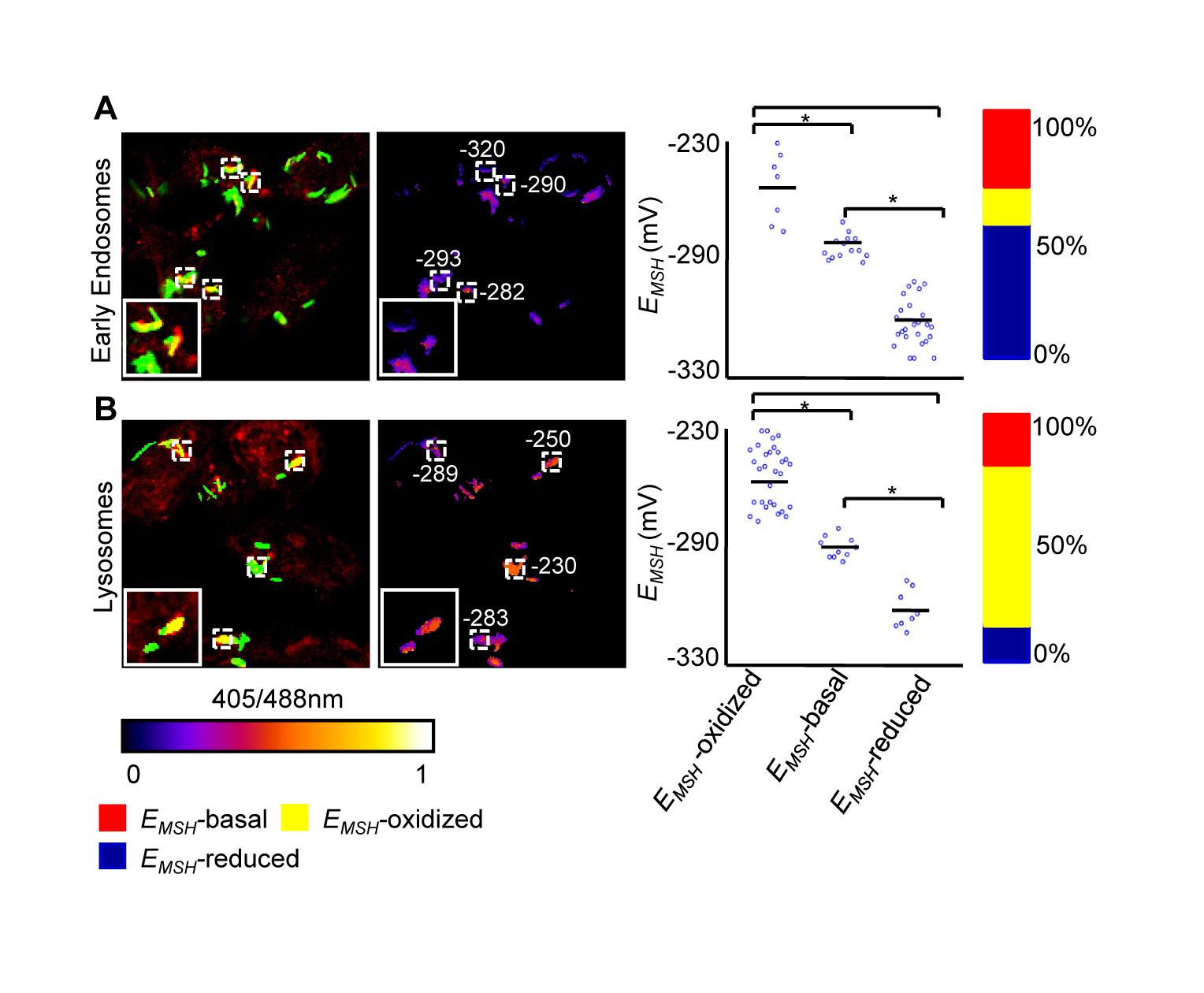

Supplement: Figure S7 — Intramycobacterial heterogeneity in EMSH within early endosomes and lysosomes. THP-1 cells were infected with H37Rv expressing Mrx1-roGFP2 (moi: 10). At each time point infected cells were treated by NEM-PFA. Cells were then stained for Rab5 and Cathepsin D and analyzed by confocal microscopy for measuring ratiometric sensor response in Mtb co-localized within sub-vacuolar compartments. Co-localization of Mtb H37Rv in (A) endosomes and (B) lysosomes. In the merge panel, green and red indicates the bacilli and the compartment markers, respectively. The overlap is demonstrated in the merge images, where yellow indicates a positive correlation. False color ratio images were generated as described in SI Materials and Methods . Small dashed line boxes indicate co-localized bacilli and large solid line boxes represent the enlarged view of the one of the co-localized bacilli. Numbers represent EMSH in millivolts. EMSH of co-localized bacilli (≥50) is calculated and distribution is shown in scatter plot. Each point on the plot represents a bacterium. Bar represents mean values. p-values were calculated by one way ANOVA followed by Tukey's HSD statistical test (* p<0.01). Percentage of bacilli in each subpopulation is represented as a stacked bar graph. Color bar corresponds to the 405/488 nm ratios ranging from 0 to 1. Data shown is the representative of at least three independent experiments. (TIF) [file ppat.1003902.s007.tif]

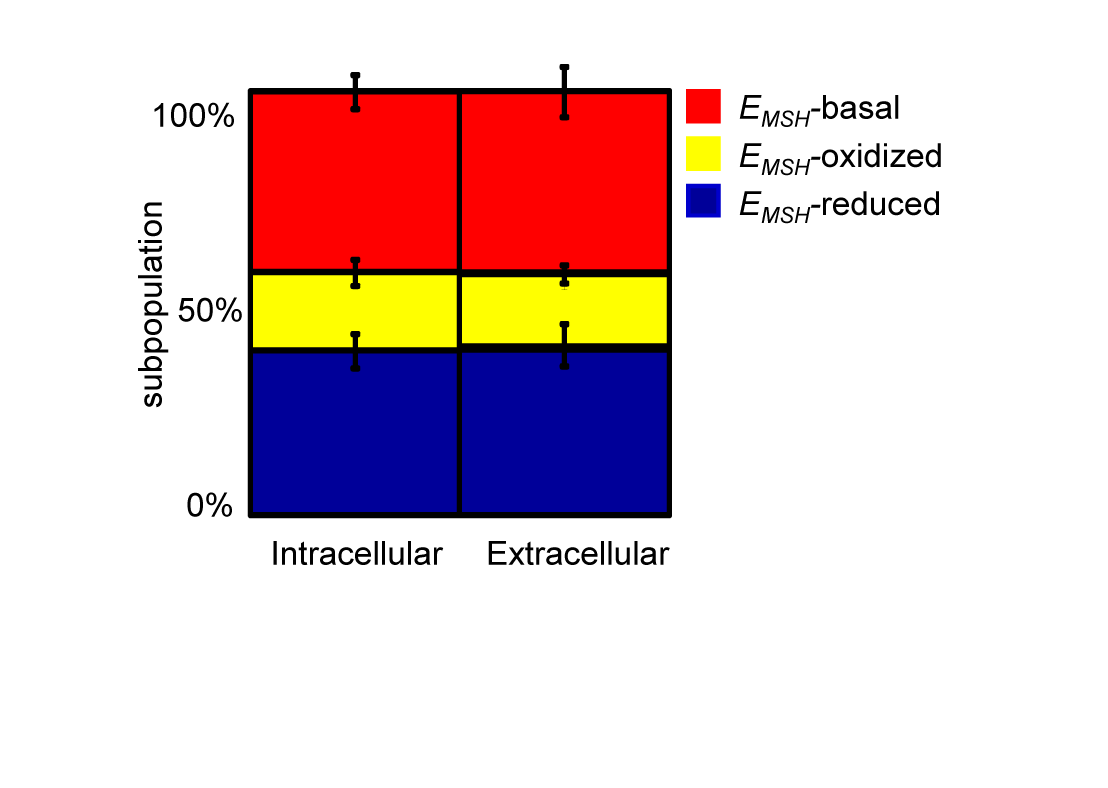

Supplement: Figure S8 — THP-1 cells were infected with H37Rv expressing Mrx1-roGFP2. 24 h p.i., cells were treated with NEM and PFA, followed by flow cytometry. In a parallel experiment, infected macrophages were lysed and redox heterogeneity within released Mtb cells was analyzed by flow cytometry. This result clearly demonstrates that NEM-treated Mtb maintains macrophage induced heterogeneity in EMSH after its liberation from macrophages. Although we have effectively blocked Mrx1-roGFP2 redox state by NEM, previous studies showed that host-induced antibiotic tolerance was preserved in Mtb even after its release from macrophages and subsequent culturing in 7H9 medium in vitro [49]. Similarly, we and others have previously reported that Mtb maintains host-induced changes in metabolism and redox state in 7H9 medium for a few generations [10], [69]. This allowed us to stain macrophage conditioned Mtb with Pi for determining membrane integrity status post-antibiotic treatment. (TIF) [file ppat.1003902.s008.tif]

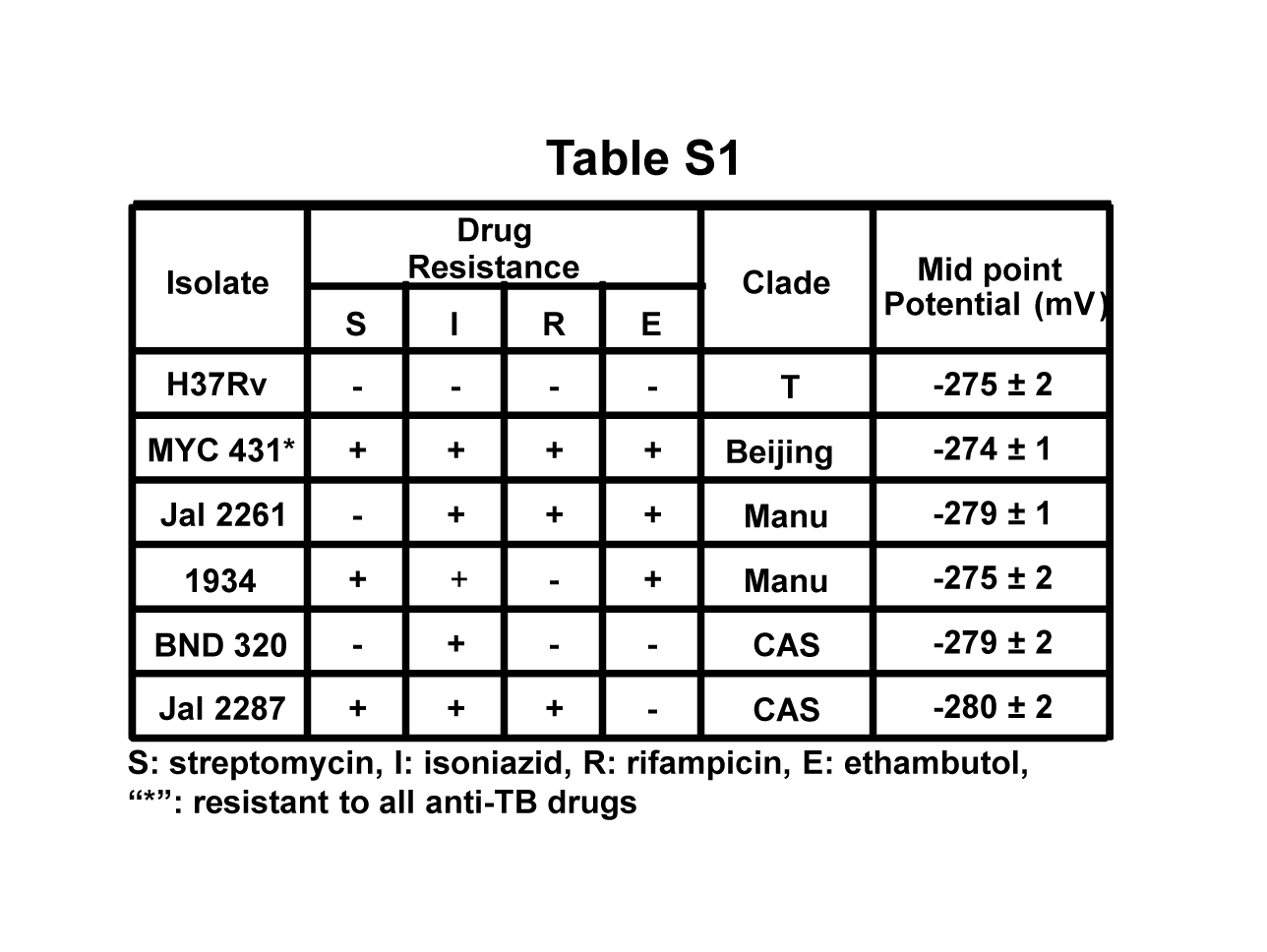

Supplement: Table S1 — Drug-resistance patterns, clade identity, and EMSH for H37Rv and different field isolates used in this study. (TIF) [file ppat.1003902.s009.tif]
