# Supplementary material for: Reengineering Redox Sensitive GFP to Measure Mycothiol Redox Potential of Mycobacterium tuberculosis during Infection
Source: PLoS Pathog. 2014 Jan 30;10(1):e1003902. doi: 10.1371/journal.ppat.1003902 (PMC3907381; doi:10.1371/journal.ppat.1003902)
Supplement: Text S1 — Supporting text. This file contains detailed methods, including in vitro redox calibration curve using Mrx1-roGFP2 protein, culture conditions for mammalian and bacterial cells, macrophage infection, quantification of Mrx1-roGFP2 expression, in vitro redox calibration curve using Mtb H37Rv, measurement of EMSH by confocal and flow cytometry, Alamar blue and growth assays, Pi staining, effect of rapamycin and DTT on anti-TB drugs activity, and statistical analysis. (DOCX) [file ppat.1003902.s010.docx]

**Supporting Information**

**Materials and Methods**

**Generation of *in vitro* redox titration curve using Mrx1-roGFP2 protein**

The redox probe was titrated in degassed PBS containing 1 µM protein and 10 mM of total DTT (varying ratios of trans-4,5-dihydroxy-1,2-dithiane (DTT_oxd_) and reduced DTT (DTT_red_)). The solutions were allowed to equilibrate for 1 h in an anaerobic glove box. Redox potential of the DTT standard solutions was calculated according to the Nernst equation:

$$E^{'}=E_{\mathrm{DTT}}^{O^{'}}-\left( \frac{\mathrm{RT}}{2F} \right)*\ln\left( \frac{\left[ \mathrm{DTT}_{\mathrm{red}} \right]}{\left[ \mathrm{DTT}_{\mathrm{oxd}} \right]} \right)$$

where E^0’^_DTT_ = -330 mV, R is the gas constant (8.313 J/mol/K), T is temperature (K), n = 2 is the number of electrons exchanged, and F is Faraday's constant (96490 J/mol/volt). The roGFP2 ratios were normalized to the values measured using 10 mM DTT_red_ as 0% oxidation and 10 mM DTT_oxd_ as 100% oxidation, and the normalized ratios were plotted against the calculated redox potentials of DTT standard solutions. Measurements were performed on spectraMaxM3 microplate reader (Molecular devices).

**Mammalian cells, bacterial cultures, and infection**

The human monocytic cell line THP-1 and mouse macrophage cell line RAW 264.7 were maintained in an atmosphere containing 5% CO_2_ at 37°C in the culture medium recommended by ATCC. THP-1 monocytes were differentiated into macrophages by a 24 h treatment with 20 ng/ml phorbol 12-myristate 13-acetate (PMA; Sigma-Aldrich Co. Saint Louis, MO, USA). Cells were rested for 3 days following chemical differentiation to ensure that they reverted to a resting phenotype before infection. RAW 264.7 macrophages were activated by treatment with 100 U/ml IFN-γ12 h before infection and 100 ng/ml LPS for 2 h before infection. The bacterial species and strains used in this study were *Msm* mc^2^155, *Msm*Δ*mshA*, *Msm*Δ*mshD*, *Msm*Δ*sigH*, *M. bovis* BCG (Pasteur), *Mtb* H37Rv and the field isolates Jal 1934, Jal 2287, Jal 2261, BND 320 and MYC 431 (kind gift from Dr. Kanury V.S. Rao, ICGEB, New Delhi). Bacteria were grown in Middlebrook 7H9 broth (Difco) supplemented with 10% OADC (Becton Dickinson), 0.1% Glycerol and 0.1% Tween 80 until the mid-log phase (OD_600_ of 0.8). Bacilli were washed twice with 10% glycerol and resuspended in 1/10^th^ the actual volume. The competent cells were electroporated using 1–2 μg of the plasmid in Bio Rad Gene Pulser with settings of 2.5 kV voltage, 25 μF capacitance and 1000 Ω resistance. After overnight recovery in 7H9, selection was performed on 7H10 agar plates containing hygromycin (50 μg/ml). After 21 days of selection, bacteria were grown in 7H9 broth till mid-log phase and used for further studies.

RAW 264.7 macrophages or PMA-differentiated THP-1 cells seeded at 2 ×10^5^ cells per well in 24-well plates were infected with Mrx1-roGFP2 expressing *Mtb* at moi of 10 and incubated for 4 h at 37°C in 5%CO_2_. Extracellular bacteria were removed by washing twice with PBS.

**Quantification of Mrx1-roGFP2 expression in *Msm***

Purified Mrx1-roGFP2 protein standards (100 to 1000 ng) were subjected to SDS-PAGE and proteins were detected by probing the blots with anti-GFP polyclonal antibodies. The scanned blots were quantified using the ImageJ software and a linear standard curve was obtained from the intensities of Mrx1-roGFP2 bands. 10 ml of *Msm* cells over expressing Mrx1-roGFP2 were grown till an OD_600_ of 0.8, harvested and resuspended in 1 ml lysis buffer (0.3 M NaCl, 20 mM sodium phosphate buffer pH 7.5, 10% glycerol and 1X protease inhibitor). 15 µg of cell lysate was subjected to SDS-PAGE followed by immuno-blotting. Quantification of expression levels was estimated from the standard curve and concentration in molar value was calculated by taking bacterial cell volume equivalent to 1x 10^-15^ liter.

***In vitro* redox calibration curve using *Mtb* H37Rv**

For estimating *E_MSH_* of *Mtb* during infection, we generated a redox calibration curve using *Mtb* cells grown in 7H9 medium *in vitro*. Because 7H9 grown *Mtb* does not show redox variations, exposure of cells to different ratios of oxidant and reductant would have uniform influence on the intracellular redox state of *Mtb*. In contrast, significant heterogeneity in *Mtb* redox state inside macrophages precludes generation of redox calibration curve *in situ*.

Absolute redox potential values in bacilli expressing Mrx1-roGFP2 were obtained from *in vitro* calibration curve generated by titrating bacilli with 1x PBS containing different ratios of DTT_oxd_ to DTT_red_ covering redox potential range of -330 to -195 mV. Mrx1-roGFP2 ratios (405/488 nm) at 510 nm emission were normalized to the range between 0% oxidation (10 mM reduced DTT) and 100% oxidation (1 mM CHP) and the normalized ratios were plotted against the calculated redox potentials of the DTT standard solution which is calculated according to the Nernst equation:

$$E^{'}=E_{\mathrm{DTT}}^{O'}-\left( \frac{\mathrm{RT}}{2F} \right)*\ln\left( \frac{\left[ \mathrm{DTT}_{\mathrm{red}} \right]}{[\mathrm{DTT}_{\mathrm{oxd}}]} \right)$$

Where E^0’^_DTT_ = -330 mV

Data were fit (Sigma plot 10 statistical analysis software) by nonlinear regression to generate a calibration curve that was then used to relate the Mrx1-roGFP2 ratios obtained from flow cytometry and/or confocal microscopy to midpoint potential in mV. Briefly, at the end of each experiment, the Mrx1-roGFP2 ratios were normalized with the ratios obtained by treatment with 10 mM DTT_red_ (0% oxidation) and with 1 mM CHP (100% oxidation). Normalized ratios were averaged and converted to redox potential (mV) by fitting the values in the calibration curve.

**Confocal microscopy**

Infected THP-1 cells were treated with 10 mM NEM, fixed with 4% PFA and permeabilized with 0.2% (w/v) Triton X-100 in 1xPBS for 20 min. Cells were blocked with 3% (w/v) BSA and 0.5% Tween 20 in 1xPBS for 1 h. For marking early endosomes, cells were stained with anti EEA1 and anti Rab5 antibodies (Santa Cruz). For staining lysosomes, prior to fixing, the cells were pre-treated for 1 h with Lysotracker (Invitrogen, 100 nM). Lysosomes were also stained with anti-Cathepsin D antibodies (Santa Cruz). For autophagosomes, prior to fixing, cells were pre-treated with E64d and pepstatin A (Sigma, 10 µg/ml each) followed by staining with primary LC3 antibody (Cell Signaling Technology). For EEA1, Rab5, Cathepsin D and LC3 staining, cells were further stained with secondary (Alexa fluor 568) antibody for 1h. The coverslips were washed thoroughly with PBS and mounted onto glass slides with mounting media (Antifade reagent, Invitrogen Molecular Probes, Carlsbad, CA, USA). Co-localizations of compartment specific markers with bacteria were determined using unbiased counting and assessment of morphological characteristics based on published guidelines [[1](#_ENREF_1),[2](#_ENREF_2)]. For measurement purposes, co-localization events were determined by evaluating ~100 bacteria from a minimum of seven random fields and processed further for calculating *E_MSH_*. Images were acquired randomly from each set with a Nikon EclipseTi-E laser-scanning confocal microscope equipped with a 60X/1.4 numerical aperture oil Plan-Apochromat differential interference contrast objective lens using the blue diode laser (excitation at 405 nm and emission at 500/530 nm), argon laser (excitation at 488 nm and emission at 500/530 nm) and helium neon laser (excitation at 543 nm and emission at 567/642 nm). Images were saved as 16-bit TIF files and analyzed by ImageJ software (http://rsb.info.nih.gov/ij/). After subtracting the background, images were converted to 32-bit format. The intensities of the 488nm images were threshold and ratio images were created by dividing the 405 nm image by the 488 nm image pixel by pixel and displayed in false colors using the lookup table "Fire".

**Preparation of *Mtb* cells for *E****_MSH_* **measurements by Flow Cytometry.**

To study *E_MSH_* of *Mtb* *in vitro* or *ex vivo*, *Mtb* grown in 7H9 or *Mtb* infected macrophages were washed with 1xPBS and treated with 10 mM NEM for 5 min at RT followed by fixation with 4% PFA for 15 min at RT. After washing thrice with PBS, bacilli or cells were scraped and analyzed using a BD FACS Verse Flow cytometer (BD Biosciences). The ratio of emission (510/10 nm) after excitation at 405 and 488 nm was calculated. Data was analyzed using the FACSuite software.

**Redox potential measurement**

The intracellular redox potential measurements were done as given in a study on Grx1-roGFP2 [[3](#_ENREF_3)]. Briefly, mycobacterial strains grown till an OD_600_ nm of 0.6 to 0.8 were harvested, washed twice and resuspended in PBS. Fluorescence excitation scan (350-500 nm) was performed at 510 nm emission. For each experiment the minimal and maximal fluorescence ratios were also determined, which correspond to 100% sensor reduction and 100% sensor oxidation, respectively. Diamide (400 µM) was used as the oxidant and DTT (40 mM) as the reductant. In case of *Mtb* cumene hydroperoxide (CHP, 1 mM) was used instead of diamide. The observed fluorescence ratio was used to calculate the corresponding degree of sensor oxidation using the equation given below.

$${OxD}_{roGFP2}=\frac{R-R_{red}}{\left( I490min/I490max \right)\left( R_{ox}-R \right)+(R-R_{red})}$$

R is the observed ratio, R_red_ and R_ox_ are the ratios of completely reduced and oxidized roGFP2, respectively. *I* 490min and *I* 490max are the fluorescence intensities measured with excitation at 490 nm for fully oxidized and fully reduced roGFP2, respectively.

Next, the intracellular sensor redox potential *E_roGFP2_* was calculated using the Nernst equation:

$$E_{roGFP2}=E_{roGFP2}^{O'}-\left( \frac{\mathrm{RT}}{2F} \right)*\ln\left( \frac{(1-\mathrm{OxD}_{roGFP2}}{\mathrm{OxD}_{roGFP2}} \right)$$

roGFP2 has an average consensus midpoint redox potential of E^0^’_roGFP2_ = -280 mV [[4](#_ENREF_4)]. Based on the equilibration between the sensor and the mycothiol redox couple we obtain the mycothiol redox potential *E_MSH_ = E_roGFP2_*.

**Alamar Blue assay and growth assay**

Metabolic activity of *Mtb* strain expressing Mrx1-roGFP2 was monitored using widely used oxidation/reduction indicator Alamar blue redox dye. A change from non-fluorescent blue to fluorescent pink color indicates reduction of Alamar Blue. The intensity of the pink color directly correlates with the extent of bacterial growth and can be easily measured by spectrofluorometry. Alamar blue assay was performed in 96 well flat bottom plates. Wt *Mtb* and Mrx1-roGFP2 harboring strain were cultured in 7H9-ADS medium and grown till exponential phase (O.D _600_ nm ~0.8). Approximately 1×10^5^ bacteria were taken per well in a total volume of 200 µl of 7H9-ADS medium. Wells containing no *Mtb* were the autofluorescence control. Additional controls consisted of wells containing cells and medium only.  After 5 days of incubation at 37ºC, 20 µl of 10X Alamar blue was added and plates were re-incubated for 24 h and the fluorescence readings were recorded. Fluorescence intensity was measured in a SpectraMax M3 plate reader (Molecular Device) in top-reading mode with excitation at 530 nm and emission at 590 nm.

To study the effect of Mrx1-roGFP2 overexpression on mycobacterial growth in liquid medium, wt *Mtb* and Mrx1-roGFP2 harboring strain were cultured in 7H9 medium supplemented with 1XOADC, 0.05% glycerol, and 0.1% Tween 80 until mid-log phase. Cells were harvested by centrifugation and washed twice with 7H9 medium without any supplement (basal). These strains were diluted to a starting O*.*D _600_ nm of 0.1-0.15 in 7H9 medium containing 1XOADC, 0.05% glycerol, and 0.1% Tween 80 at incubated at 37ºC in roller culture bottle at 2 rpm . At each time point, an aliquot from each strain was removed, lightly sonicated, and the O*.*D _600_ nm was measured.

**Pi staining**

For Pi staining, redox state of bacilli was first blocked by treatment with NEM followed by macrophage lysis by PBS containing 0.01% SDS. Released bacilli were stained with 10 µM propidium iodide (Pi) for 15 min at RT in dark. After washing twice with 1x PBS, bacilli were fixed in PFA, washed again with PBS and analyzed by flow cytometer.

**Antimicrobial activity of drugs upon treatment with rapamycin and DTT**

To study the effect of autophagy on antibiotic-mediated redox stress and killing, non lethal concentration of rapamycin (200nM) was used along with INH and CFZ. Briefly, THP-1 cells infected with H37Rv were treated with INH (0.5 µg/ml) and CFZ (0.5 µg/ml) with or without rapamycin. 24 h p.i., macrophages were lysed and bacilli were stained with Pi followed by flow cytometry.

To investigate the effect of DTT on antibiotics efficacy, we treated exponential phase (OD _600_ nm of 0.5) grown *Mtb* H37Rv with 5X MIC of INH, CFZ, RIF and ETH in the presence or absence of 5 mM of DTT. DTT was added at 0 and 2 day post antibiotic-exposure. After incubation for 4 days, bacilli were plated for CFU.

**Statistical analysis**

All data were derived from at least three independent experiments. Statistical analyses were conducted using Graphpad Prism software and values were presented as mean ± SD. The statistical significance of the differences between experimental groups was determined by two-tailed, unpaired student’s *t*-test unless specified. Differences with a p value of <0.05 were considered significant. Statistical significance between subpopulations of *Mtb* with different *E_MSH_* was calculated by one way ANOVA followed by tukey's HSD statistical test (* p<0.01).

**References:**

1. Fratti RA, Backer JM, Gruenberg J, Corvera S, Deretic V (2001) Role of phosphatidylinositol 3-kinase and Rab5 effectors in phagosomal biogenesis and mycobacterial phagosome maturation arrest. J Cell Biol 154: 631-644.

2. Fratti RA, Chua J, Vergne I, Deretic V (2003) Mycobacterium tuberculosis glycosylated phosphatidylinositol causes phagosome maturation arrest. Proc Natl Acad Sci U S A 100: 5437-5442.

3. Gutscher M, Pauleau AL, Marty L, Brach T, Wabnitz GH, et al. (2008) Real-time imaging of the intracellular glutathione redox potential. Nat Methods 5: 553-559.

4. Hanson GT, Aggeler R, Oglesbee D, Cannon M, Capaldi RA, et al. (2004) Investigating mitochondrial redox potential with redox-sensitive green fluorescent protein indicators. J Biol Chem 279: 13044-13053.
